# Effectiveness of interventions to indirectly support food and drink intake in people with dementia: Eating and Drinking Well IN dementiA (EDWINA) systematic review

**DOI:** 10.1186/s12877-016-0256-8

**Published:** 2016-05-04

**Authors:** Diane K. Bunn, Asmaa Abdelhamid, Maddie Copley, Vicky Cowap, Angela Dickinson, Amanda Howe, Anne Killett, Fiona Poland, John F. Potter, Kate Richardson, David Smithard, Chris Fox, Lee Hooper

**Affiliations:** Norwich Medical School, University of East Anglia, Norwich Research Park, Norfolk, NR4 7TJ UK; Age UK Norfolk, 300 St Faith’s Road, Old Catton, Norwich, NR6 7BJ UK; NorseCare, Lancaster House, 16 Central Avenue, St Andrew’s Business Park, Norwich, NR7 0HR UK; School of Health and Social Work, University of Hertfordshire, Hatfield, Hertfordshire AL10 9AB UK; School of Health Sciences, University of East Anglia, Norwich Research Park, Norfolk, NR4 7TJ UK; Norfolk and Norwich University Hospital, Colney Lane, Norwich, NR4 7UY UK; King’s College Hospital NHS Foundation Trust, Denmark Hill, London, SE5 9RS UK; Norfolk and Suffolk NHS Foundation Trust, Hellesdon Hospital, Drayton High Road, Norwich, NR6 5BE UK; Present address: Royal College of Paediatrics and Child Health, 5-11 Theobalds Road, London, WC1X 8SH UK

**Keywords:** Dementia, Aged, Eating, Drinking, Meta-analysis, Diet, Malnutrition, Dehydration

## Abstract

**Background:**

Risks and prevalence of malnutrition and dehydration are high in older people but even higher in older people with dementia. In the EDWINA (Eating and Drinking Well IN dementiA) systematic review we aimed to assess effectiveness of interventions aiming to improve, maintain or facilitate food/drink intake indirectly, through food service or dining environment modification, education, exercise or behavioural interventions in people with cognitive impairment or dementia (across all settings, levels of care and support, types and degrees of dementia).

**Methods:**

We comprehensively searched Medline and twelve further databases, plus bibliographies, for intervention studies with ≥3 cognitively impaired adult participants (any type/stage). The review was conducted with service user input in accordance with Cochrane Collaboration’s guidelines. We duplicated assessment of inclusion, data extraction, and validity assessment, tabulating data. Meta-analysis (statistical pooling) was not appropriate so data were tabulated and synthesised narratively.

**Results:**

We included 56 interventions (reported in 51 studies). Studies were small and there were no clearly effective, or clearly ineffective, interventions. Promising interventions included: eating meals with care-givers, family style meals, soothing mealtime music, constantly accessible snacks and longer mealtimes, education and support for formal and informal care-givers, spaced retrieval and Montessori activities, facilitated breakfast clubs, multisensory exercise and multicomponent interventions.

**Conclusions:**

We found no definitive evidence on effectiveness, or lack of effectiveness, of specific interventions but studies were small and short term. A variety of promising indirect interventions need to be tested in large, high-quality RCTs, and may be approaches that people with dementia and their formal or informal care-givers would wish to try.

**Trial registration:**

The systematic review protocol was registered (CRD42014007611) and is published, with the full MEDLINE search strategy, on Prospero (http://www.crd.york.ac.uk/PROSPERO/display_record.asp?ID=CRD42014007611).

**Electronic supplementary material:**

The online version of this article (doi:10.1186/s12877-016-0256-8) contains supplementary material, which is available to authorized users.

## Background

Dementia is becoming one of the most pressing challenges for the care of older people, and the main contributor to disability and dependence. Half of older people needing personal care have dementia, rising to 80 % of those living in nursing homes [1]. Risks for, and prevalence of, malnutrition and dehydration are high in older people but even higher in older people with dementia [2, 3].

Interventions to support older people around eating and drinking vary: changing the colour of a plate, increasing exercise, altering the environment or changing knowledge or attitudes, [4] and the full range of interventions may be helpful for people with dementia. Some interventions alter food/drink directly (via oral nutrition supplementation, food modification, dysphagia management, eating assistance and/or supporting the social element of eating and drinking) and some aim to affect food/drink intake or experience indirectly (altering dining environment or food service, providing education or training for people with dementia or their care-givers, behavioural interventions, exercise programs, and combinations of these, multicomponent interventions).

An international survey aiming to establish research priorities in nursing homes found both dementia care and nutrition to be crucial areas of exploration [5]. This systematic review paper reports on indirect interventions, and its partner report assessed direct interventions [6]. Together they provide the underpinning research evidence by systematically reviewing existing research on all interventions aiming to improve, maintain or facilitate food or drink intake (directly or indirectly) in adults with dementia of any stage and in any setting.

In this systematic review we have used Cochrane terminology. The term “systematic review” means the whole process of specifying a clear question, searching for relevant studies, assessing whether they meet inclusion criteria, data extracting those that do, assessing validity, and reporting the findings [7]. Systematic reviews may contain statistical pooling, called meta-analysis [7].

## Methods

We developed the systematic review protocol collaboratively, and the review team included lay stakeholders, subject experts and methodological experts. Lay stakeholders included members from AgeUK Norfolk and NorseCare (residential homes group). We also worked with two patient and public involvement groups (the Public & Patient Involvement in Research, PPIRes, from Norfolk and Suffolk and the Public Involvement in Research Group, PIRG, from the University of Hertfordshire) to develop additional specific questions for the review. The protocol is published, with the full MEDLINE search strategy, on Prospero [8]. The review was conducted in accordance with Cochrane Collaboration’s guidelines, [7] and reported in accordance with PRISMA guidance [9]. Study methods and specific questions posed by patient and public groups are reported in full (Additional file 1), and summarised below.

### Criteria for inclusion

We included randomised (RCTs) and non-randomised (CCTs, with a concurrent but non-randomised control group) controlled trials and before/after (BA or pre-post) studies that fulfilled the following criteria:

- Participants: ≥3 adults (to eliminate case reports which cannot be assumed to be generalisable) diagnosed with any type/stage of dementia or mild cognitive impairment (MCI) or where the mean Mini Mental State Examination (MMSE) score plus one standard deviation was ≤26, in any setting.
- Duration: ≥5 consecutive days (intake at a single meal or snack or over a short period of time has little overall effect on nutritional status, so we limited to ≥5 consecutive days to suggest longer term changes and patterns which may affect nutritional status).
- Intervention: aimed to indirectly alter nutrition or hydration status, food, drink or nutrient intake or increase meaningful activity by altering the dining environment or food service, providing education or training of people with dementia or their care-givers, providing a behavioural intervention, exercise, or a multicomponent intervention (>3 interventions, including at least one listed here).
- Primary outcomes: nutrition or hydration status, [10] meaningful activity or enjoyment of food or drink (activity around food or drink that is personally fulfilling, that people enjoy, look forward to or find important), quality of life. Secondary outcomes: quantity, quality or adequacy of food or fluid intake (including ability to eat independently, and swallow without aspirating). Note - studies were only included if they collected at least one of these outcomes, but where studies were included we also extracted, and report, data provided on the following outcomes: functional or cognitive status, views or attitudes, cost effectiveness, resource use, mortality and health outcomes.

### Search strategy

We developed a complex MEDLINE search strategy and adapted it for 12 further databases (EMBASE, CINAHL, PsychInfo, five Cochrane Databases, meta-register of controlled trials, ALOIS (Cochrane Dementia and Cognitive Improvement Group comprehensive register of dementia trials), Dissertation and Thesis abstracts, and International Alzheimer’s Disease Research Portfolio (IADRP) from inception to March 2014, without language or date limitations. Bibliographies of included studies and lists of included/excluded studies from relevant reviews were checked [11–17].

### Study selection and data collection

Inclusion was assessed by two reviewers independently using an inclusion form. Data (publication details, participants, intervention, comparison, outcomes as above plus quality of life, functional or cognitive status, views or attitudes, cost effectiveness, resource use, mortality, health outcomes) and validity characteristics were extracted independently in duplicate. Methodological quality was assessed using Cochrane risk of bias tool [18]. In addition to generic criteria, we assessed funding bias, validity of dementia diagnosis, outcome measures and baseline comparability between groups. We considered a study at low risk of bias where it was at low risk of both selection bias (was randomised and had appropriate allocation concealment) and detection bias (blinding of outcome assessment).

### Data synthesis

Studies were grouped by type of intervention then study design for tables and narrative synthesis. Type of intervention included:

1. Dining environment and food service which included any alteration to the physical environment in which food and/or drink was taken. This included furniture, noise levels and other sensory adjustments or any alteration to the manner in which food was served, including coloured tableware, waitress service.
2. Education/training which included interventions with an educational and/or awareness component for people with dementia and/or their formal or informal care-givers
3. Behavioural interventions were interventions that aimed to alter the behaviour of people with dementia, such as verbal prompting or relaxing music prior to a meal.
4. Exercise was any intervention with an exercise component.
5. Multicomponent interventions included ≥3 intervention components, including at least one of those above.

Random-effects meta-analysis of RCTs using Review Manager (RevMan 5.3) software was planned where studies were suitably comparable, and narrative comparison was planned for all study types and to address questions formulated by the public (Table 1).

**Table 1:** Specific review questions formulated by members of the Public & Patient Involvement Groups, and the evidence found to address these questions. What are the most effective ways to encourage people with dementia to eat, drink and maintain nutritional intake? Information provided here is supplemental to the main findings of this review, and overall evidence is weak or lacking – the review does not definitively show that any intervention is either useful or not useful

Area	Questions from lay stakeholders	Review findings
*1. Type of dementia*	For people with different types of dementia (Alzheimer’s, vascular, dementia with Lewy bodies, other types or mixed types), what interventions can help to maintain or improve food intake or nutritional status and fluid intake or hydration status?	*Not all interventions reported the type of dementia or cognitive impairment, but those that did enrolled people with AD or a mixture of people with AD and other dementias. There was no reason to suggest that effects of interventions in people with AD were different from those in people with mixed dementia, but more research is needed to clarify.*
*2. Stage of dementia*	What interventions can help to maintain or improve food intake or nutritional status and fluid intake or hydration status in people with mild cognitive impairment, mild/moderate/severe dementia?	*Exercise and multicomponent interventions did not usually specify dementia severity. * *MCI: One intervention assessed effects of resident and staff education for 269 people with MCI living in an old age hostel, finding no effects on weight or cognition (Kwok 2012).* *Mild to moderate dementia: few interventions of dining environment and food service interventions included people with mild dementia. Educational interventions for formal care-givers included people with mild to moderate dementia but effects appeared to depend on the intensity of education and support, rather than degree of dementia of participants, with only the most intensive intervention appearing useful (Mamhidir 2007). Reminiscence cooking and a supported breakfast club, both interventions supporting social interaction, appeared to promote meaningful involvement in people with mild to moderate dementia (Santo Pietro 1998, Huang 2009).* *Moderate to severe dementia: most dining environment and food service interventions included people with moderate to severe dementia, so results for these interventions are likely to apply to people with moderate to severe dementia. Educational interventions for formal care-givers included people with moderate to severe dementia but effects appeared to depend on the intensity of education and support, rather than degree of dementia of participants, with only the most intensive intervention appearing useful (Mamhidir 2007). Behavioural interventions in people with severe dementia appeared to promote eating independence, without improving nutritional status (Van Ort 1995, Coyne 1998, Beattie 2004).*
*3. Setting*	• For people with dementia living in residential care or residing in a medical setting, what interventions can help to maintain or improve food intake or nutritional status and fluid intake or hydration status? • For people with dementia living in their own homes with or without a care-giver (full-time or occasional; close relative or paid care-giver), what interventions can help to maintain or improve food intake or nutritional status and fluid intake or hydration status?	*Most of the studies were conducted in various residential or nursing settings, and very few in participants own homes. Generally, effectiveness of interventions related to the effectiveness of interventions in residential settings. For people with dementia living at home nutritional education of caregivers and people with dementia appeared useful in supporting weight in one study (Riviere 2001), but not in two others (Suominen 2013, NutriAlz Trial).*
*4. Emotional & social issues*	For people with dementia, does emotional closeness of the care-giver (e.g. close relative vs paid care-giver) affect the outcomes?	*Emotional closeness to the care-giver was not ever reported, and in most interventions care-givers appeared to be professional rather than family care-givers (also see “Setting”).*
*5. Meaningful activity*	• For people with dementia, what interventions aimed at improving or maintaining food and/or fluid intake, nutrition or hydration status, support meaningful activity (activity around food or drink that is personally fulfilling, that people enjoy, look forward to or find important)? • For people with dementia, are there any interventions that decrease food or fluid intake, diminish enjoyment or quality of life, or diminish meaningful activity or social inclusion?	*Few studies measured quality of life or happiness using a validated scale, but some reported improved autonomy, involvement and interest of participants. There were suggestions that music at dinnertime might improve psychological wellbeing (Ragneskog 1996), familiar lunchtime music might increase social engagement (Thomas 2009), family style meals with staff training might improve mealtime participation (Altus 2002), nutritional education for people with dementia and their spouses living at home might improve quality of life (Suominen 2013), reminiscence cooking might improve happiness and feelings of participation (Huang 2009), and a facilitated breakfast club improve interest and involvement (Santo Pietro 1998). Fingerfoods, verbal prompting and positive reinforcement, behavioural interventions (spaced retrieval and Montessori activities), adapted Tai-Chi and cognition action exercise may improve eating independence (Jean 1997, Coyne 1988, Van Ort 1995, Lin 2010, 2011, Dechamps 2010).*
*6. Individualised interventions*	Do individualised interventions appear more effective than those that are not individualised, in helping people with dementia to maintain or improve food and/or drink intake, nutrition or hydration status (or related outcomes)?	*Only a few interventions were individualised (Mentes 2003, Suominen 2007 and 2013, Kwok 2012, Huang 2009, Wu 2013, Rolland 2007, Beck 2010, Boffelli 2004 and Keller 2003), but these did not stand out as being more effective than others. One study directly compared a fixed intervention (spaced retrieval training combined with Montessori activities over 24 sessions) with an individualised approach (as the fixed intervention but with different sessions adapted to each participants learning response), and a control arm (Wu 2013). There were no clear differences between the arms: BMI improved in both fixed and individualised interventions, but depression was only reduced in the individualised arm.*
*7. Interventions in acute illness*	Are there any interventions that are particularly effective in helping people with dementia to maintain or improve food and/or drink intake, nutrition or hydration status (or related outcomes) during periods of acute illness?	*None of these interventions were assessed on people who were acutely ill.*

## Results

**Fig. 1:**
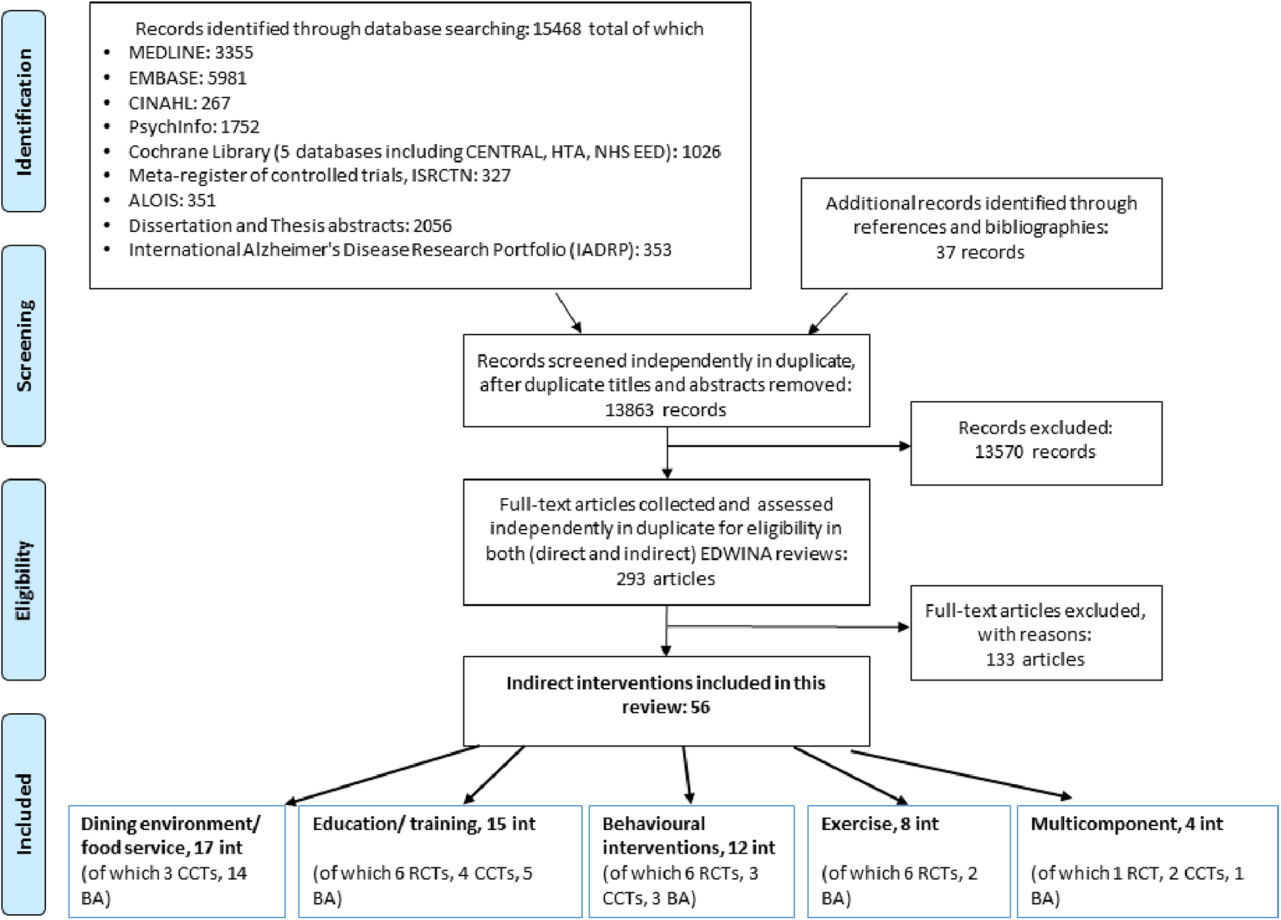
EDWINA systematic review PRISMA flow diagram for studies of indirect interventions

Electronic searches identified 15,468 citations, with a further 37 from bibliographies. After de-duplication we assessed 13,863 titles and abstracts, and collected 293 full text papers for further assessment. Fifty one studies reporting on 56 interventions were included in this review (Fig. 1).

Brief characteristics and results of the included interventions are shown in Tables 2, 3, 4, 5 and 6, with fuller details in Additional file 2. Most interventions were tested in North America (29 interventions) with 16 tested in Europe, eight in Asia, two in New Zealand and one in South America. The majority of interventions were assessed in institutional settings (17 in dementia units, 15 in nursing homes or units, 11 in long-term care, two in a mixture of institutional settings, six in other institutional settings, two in day-care, two living at home in the community and one with an unclear setting).

**Table 2:** Summary of characteristics and results of 17 included interventions (reported in 15 studies) investigating dining environment and food service (for further detail see Additional file 2)

Study	Design	Setting, intervention type	No.	Dementia diagnosed	Dementia stage	Dementia type	Nutrition/hydration effect	Intake effect	Quality effect (including QoL or meaningful activity) and other outcomes	Duration
Altus 2002 [19] Period 1 USA	BA	Locked dementia unit. Family-style meals	I = 5 C = NR	Yes	Mod-severe	AD & others	NR	NR	? Resident mealtime participation, ? Communication during meals, ? Praise by nurse (all improved but statistical significance unclear)	5 days
Brush 2002 [20] USA	BA	2 LTC facilities. Improved dining room lighting and table setting contrast	25	Yes	Mod-severe	NR	NR	↑ E intake	→ Quality & adequacy of food intake, → Functional status	4 weeks
Charras 2010 [21] France	CCT	Dementia units in nursing homes. Shared mealtime with staff	18	Yes	Severe	AD	↑ Weight	NR	? Greater autonomy, helping with serving and clearing up, eating independently ? Increased and higher quality resident-resident and resident-staff interaction ? Longer meals ? Better food quality ? Greater staff satisfaction (improvements based on reported observations, no significance testing)	6 months
Desai 2007 [22] Canada	CCT	2 LTC facilities, Bulk food service and home-like setting	I = 22 C = 26	Yes	NR	AD	? BMI	↑ E intake ↑ CHO intake ↑ Protein intake	NR	3 weeks
Dunne, 2004 [23] Study 1 USA	BA	LTC unit. High & low contrast red tableware	9	Yes	Severe	AD	NR	→ % Food intake ↑ % Fluid intake	NR	10 days
Dunne, 2004 [23] Study 2 USA	BA	LTC unit. High & low contrast tableware (3 conditions)	9	Yes	Severe	AD	NR	→ % Food intake → % Fluid intake	NR	10 days each
Edwards 2013 [24] USA	BA	Specialised dementia units. Dining area aquarium	70	Yes	severe	NR	→ Weight*	↑ Quantity of food & drink intake	NR	8 weeks
Kenkmann 2010 [25, 79] UK	CCT	6 Care homes. Improved dining environment & atmosphere, available snacks and drinks machines, increased food choice, extended restaurant hours	I = 57 C = 48	NR	NR	NR	→ Weight → BMI → Appears hydrated	NR	→ Enjoyment of food and drink → Cognition	1 year
Koss 1998 [26] USA	BA	High functioning dementia unit. Dining environment enhanced lighting and contrast	13	Yes	NR	AD	NR	→ Quantity of food intake	NR	3 weeks
McDaniel 2001 [27] USA	BA	Dementia unit. Large bright cafeteria style dining room vs small darker room with relaxing music	16	Yes	Various	AD	→ Weight	→ E intake → Fluid intake	NR	2 weeks
Perivolaris 2006 [29] Period 1 Canada	BA	LTC facility. Enhanced dining (small welcoming dining rooms, music, bread & coffee aroma, menu board, staff using cues and prompts)	11	Yes	Mod-severe	Various	NR	↑ E intake	→ Feeding ability → Agitation level → Resident satisfaction ? Residents eating more leisurely, less wandering, more relaxed (according to staff notes from focus group)	6 weeks
Ragneskog 1996 [30, 80] Sweden	BA	Nursing home. Dinner music (soothing music, familiar tunes, pop music)	20	Yes	Mod-severe	Various	NR	? Weight ↑ Food quantity (pop music) → Food quantity (familiar & soothing music)	↑ Psychological wellbeing → Motor impairment → Intellectual impairment → Emotional impairment ? more time taken for meal	8–10 days each
Shatenstein 2000 [31] Canada	BA	Dementia unit. Decentralised food service	22	Yes	NR	AD & others	→ Weight, → BMI, → TST → AC ↓ Albumin	↑ % food intake, ↑ E intake, ↑ CHO intake, ↑ Protein intake	NR	10 weeks
Thomas 2009 [32] USA	BA	Nursing home. Lunchtime music (variety of styles but familiar to participants)	12	Yes	Mod	AD	NR	? Quantity of food intake	? Anecdotal reports of increased social engagement, remaining in dining area longer, responding to music with dancing, foot tapping etc.	8 weeks
Van Ort 1995 [28] (contextual intervention) USA	BA	Secure nursing unit. Improved dining environment (protected mealtimes, noise & distractions minimised, meals taken in dining area, seated at tables, finger foods provided)	7	Yes	Severe	NR	→ Weight	? Quantity consumed	? Greater self-feeding behaviour ? Meals did not take longer ? Those with milder dementia received more food and interacted more with their care-givers	2 weeks
Wong 2008 [33] Period 2 New Zealand	BA	Short stay assessment unit. 24 h snacks and earlier meals	40	Yes	NR	NR	↑ BMI → AC	? E intake	NR	12 weeks
Wong 2008 [33] Period 4 New Zealand	BA	Short stay assessment unit. Mealtime soothing music	28	Yes	NR	NR	↑ BMI → AC	? E intake	NR	12 weeks

*Calculated *P* value = 0.65 but paper reports significant t-test results

*AC* various measures of arm circumference, *AD* Alzheimer’s Disease, *BA* before after (pre-post) study, *BMI* body mass index, *CCT* clinical controlled trial, *CHO* carbohydrate, *C* control, *E* energy, *Hb* haemoglobin, *I* intervention, *ICW* intracellular water, *LTC* long term care, *MCI* mild cognitive impairment, *MMSE* mini mental state examination, *MNA* mini-nutritional assessment, *Mod* moderate, *N/A* not applicable, *NR* not reported, *ONS* oral nutrition supplement, *PEM* protein energy malnutrition, *QoL* quality of life, *RCT* randomised controlled trial, *TSF* triceps skinfold measure, *TST* triceps skinfold thickness

**Table 3:** Summary of characteristics and results of 15 included educational interventions, reported in 15 studies (for further detail see Additional file 2)

Study	Design	Setting, intervention type	No. of participants	Dementia diagnosed	Dementia stage	Dementia type	Effects on nutrition and/or hydration	Effects on intake of food and/or drink and/or nutrients	Quality effect (including QoL or meaningful activity) and other outcomes	Duration
Altus 2002 [19] (Period 2) USA	BA	Locked dementia unit. Family-style meals plus nurse training	5	Yes	Mod-severe	AD & others	NR	NR	? Suggested improvements in mealtime participation & communication, and staff praise statements, but no statistical significance provided.	5 days
Aselage 2011 [38] USA	RCT	Nursing home. Staff education in eating & feeding skills	I = 4 C = 3	Yes	Mod	NR	→ Weight	? % Food consumed	? Fall in QoL likely but significance not reported ? Eating impairment	2 months
Faxen-Irving 2002 [39] Sweden	CCT	Group-living for people with dementia. ONS & staff education vs usual care	I = 21 C = 12	Yes	Mixed	Mixed	Education + ONS: ↑ BMI ↑ Weight ↑ TSF → AC → Albumin → Hb After ONS withdrawn ↓ Weight	→ Nutrition risk	Education + ONS: → Functional status ↓ Cognition (MMSE)	5 months
Hanson 2010 [37, 81–84] USA	RCT	Nursing homes. Education of surrogates on feeding options	I = 127 C = 129	Yes	Severe	NR	↓ Weight loss	NR	? Knowledge, decisional conflict and certainty (only assessed for intervention group)	9 months
Jean 1997 [40] USA	BA	Nursing home. Finger food menu plus staff training	12	NR	NR	AD & others	? Weight loss arrest	? ONS could be withdrawn in 25 % of participants	? Feeding independence	6 months
Kwok 2012 [41] Hong Kong	RCT	Old age hostels. Resident & staff education with individual dietary counselling	I = 120 C = 149	Yes	MCI	N/A	→ Weight	→ Fruit intake → Vegetable intake ↑ fish intake	→ Cognitive status	33 months
Mamhidir 2007 [42] Sweden	CCT	Nursing homes. Substantial staff training & support in integrity-promoting care	I = 18 C = 15	Yes	Various	Various	↑ Weight	NR	? Mealtime environment & routines (Qualitative analysis of staff diaries)	3 months
Mentes 2003 [43, 85] USA	BA	Nursing homes. Hydration management staff training	8	NR	NR	NR	→ Urine specific gravity	→ Fluid intake	NR	4 weeks
NutriAlz Trial Salva 2009 [46, 86–89] Spain	RCT	Outpatient clinics and hospital day-care centres. Personalised nutrition education program for people with dementia & caregivers	I = 448 C = 498	Yes	Mild-Mod	AD, vascular & other	→ Weight, → BMI, ↑ MNA	NR	→ Eating behaviour, → Caregiver burden → Cognitive status → Functional status	12 months
Perivolaris 2006 [29] Period 2 Canada	BA	Long term care facility. Staff education (1 day workshop to assist in providing meaningful dining experience)	11	Yes	Mod-severe	Various	NR	→ E intake	→ Agitation →Eating ability → Resident satisfaction ? Eating pace more leisurely ? Less wandering ? More relaxed.	6 weeks
Pivi 2011 [44] Brazil	RCT	Unclear. People with dementia & caregiver nutritional education program	I = 25 C = 27	Yes	Mild-severe	AD	? BMI, ? Weight ? AC → TSF → Albumin	NR	NR	6 months
Riviere 2001 [45] France, Italy & Spain	CCT	Living at home with informal care-giver. Caregiver nutritional education	I = 151 C = 74	Yes	NR	AD	↑ Weight, ↑ MNA	NR	→ Functional status, ↓ Cognitive status	12 months
Suominen 2007 [47] Finland	BA	Nursing home. Staff training	21	Yes	Mod-severe	NR	→ Weight, → BMI, → MNA	↑ E intake ↑ Protein intake	? Staff reported improved confidence in assessing intake and making nutritional changes	12 months
Suominen 2013 [48, 50, 90] Finland	RCT	Community. Tailored nutritional training for people with dementia & spouses	I = 50 C = 50	Yes	NR	AD	→ Weight	↑ Protein intake	? Reported improvement in QoL	12 months
Wikby 2009 [49] Sweden	CCT	Residential care. Dietary management staff training	I = 68 C = 59	NR	NR	NR	→ Weight → PEM → TSF → AC	NR	↑ Functional status, ↑ Cognition	4 months

For abbreviations see below Table 2

**Table 4:** Summary of characteristics and results of 12 included behavioural interventions, reported in 10 studies (for further detail see Additional file 2)

Study	Design	Setting, intervention type	No. of participants	Dementia diagnosed	Dementia stage	Dementia type	Effects on nutrition and/or hydration status	Effects on intake of food, drinks and/or nutrients	Quality effect (including QoL or meaningful activity) and other outcomes	Duration
Beattie 2004 [51] USA	BA	Dementia specific unit. Behavioural conditioning	3	Yes	Severe	AD	→ Weight	→ % Food intake → % Fluid intake	NR	2 weeks (total 5 weeks)
Coyne 1988 [52] USA	RCT	Dementia unit in nursing home. Verbal prompting and positive reinforcement by staff	I = 12 C = 12	Yes	Severe	AD & others	NR	NR	↑ Eating independence for solid foods → Eating independence for liquid foods → Frequency of eating solid and liquid foods	2 weeks
Eaton 1986 [53] USA	RCT	Skilled care facility. Gentle mealtime touch & verbal prompting	I = 21 C = 21	NR	NR	NR	NR	↑ E intake ↑ Protein intake	NR	5 days
Huang 2009 [54] Taiwan	BA	Older person care facility. Reminiscence cooking therapy	12	Yes	Mild- mod	NR	NR	NR	↑ Feeling of happiness → Positive communication ? Participatory feeling → Cognitive function	8 weeks
Lin 2010 [55] Spaced retrieval Taiwan	RCT	Dementia unit. Spaced retrieval	I = 32 C = 24	Yes	Various	NR	→ Weight → BMI ↑ MNA	→ Food intake	↑ Improved eating difficulty	8 weeks
Lin 2010 [55] Montessori Taiwan	RCT	Dementia unit, Montessori activities	I = 29 C = 24	Yes	Various	NR	→ Weight → BMI → MNA	↓ Food intake	↑ Improved eating difficulty	8 weeks
Lin 2011 [56] Taiwan	RCT	Dementia unit. Montessori-based activities	29	Yes	Mild-severe	NR	→ BMI → MNA	NR	↑ Eating functional ability ↑ Eating ability → Eating time ↑ Self-feeding frequency	8 weeks
McHugh 2012 [58] USA	RCT	Memory support unit/care facility. Vocal re-creative music therapy	I = 8 C = 7	Yes	Mild-Mod	AD & others	NR	→ Proportion food eaten	? Participation	3 weeks
Santo Pietro 1998 [59] USA	CCT	Dementia unit within a nursing home. Breakfast club (communication therapy)	I = 20 C = 20	Yes	Mild-Mod	AD	NR	NR	↑ Interest &involvement, ↑ Procedural memory ? Functional status ? Cognitive status ? Used humour & empathic statements, remembered names, responded to non-verbal cues, spontaneous singing, decreased distractibility & wandering.	12 weeks
Van Ort 1995 [28] Behavioural intervention USA	BA	Secure nursing unit. Systematic prompting, cuing, behavioural guidance & reinforcement	7	Yes	Severe	NR	→ Weight	?	? Self-feeding behaviour ? Longer meal-times ? Increased independence	2 weeks
Wu 2013 [57] Fixed intervention Taiwan	CCT	Dementia unit. Spaced retrieval & Montessori activities	I = 25 C = 27	Yes	Mild-severe	NR	↑ BMI ↑ MNA	NR	→ Depression	6 months
Wu 2013 [57] Individualised intervention Taiwan	CCT	Dementia units. Individualised spaced retrieval & Montessori activities	I = 38 C = 27	Yes	Mild-severe	NR	↑ BMI ↑ MNA	NR	↓ Depression	6 months

For abbreviations see below Table 2

**Table 5:** Summary of characteristics and results of eight included exercise interventions, reported in seven studies (for further detail see Additional file 2)

Study	Design	Setting, intervention details	No. of participants	Dementia diagnosed	Dementia stage	Dementia type	Effects on nutrition and/or hydration status	Effects on intake of food, drink and/or nutrients	Quality effect (including. QoL or meaningful activity) and other outcomes	Duration
Chang 2011 [61] Taiwan	BA	Day care centre. Exercise (stretching, walking, weight bearing) with encouragement & rewards	29	Yes	NR	NR	NR	NR	→ Feeding function ↑ Functional status	4 months
Dechamps 2010 [62] Adapted Tai Chi France	RCT (3 arms)	Nursing Homes & Long term care home. Adapted tai chi	I = 51 C = 60	NR	NR	AD & others	NR	NR	↑ Feeding independence → MMSE	6 months
Dechamps 2010 [62] Cognition action France	RCT (3 arms)	Nursing Homes & Long term care home. Cognition action	I = 49 C = 60	NR	NR	AD & others	NR	NR	↑ Feeding independence → MMSE	6 months
FICSIT trial (Fiatarone 1994) [63, 91–93] USA	RCT (4 arms)	Nursing home (long term rehabilitation centre). High-intensity exercise ± ONS vs placebo activities ± ONS	Ex ± ONS 50 Control ± ONS 50	NR	NR	NR	→ Weight → Thigh muscle area → Whole body potassium	↑ E-intake → Physical activity ↑ Muscle strength & mobility	→ Mortality	10 weeks
FOPANU study [60, 94, 95] Sweden	RCT	Residential care facilities. High-intensity exercise program (+/- protein supplement) vs control activity (+/- protein supplement)	I = 83 C = 94	NR	NR	NR	↓ Weight ↓ ICW	N/A	→ Mortality → Balance ↑ Gait speed, self-paced → Gait speed, maximum ↑ Lower limb strength	3 months
Heyn 2003 [64] USA	BA	Memory care residence. Multi-sensory exercise program (focused attention, flexibility & aerobic exercise, strength training, relaxation & breathing techniques)	13	Yes	Mostly severe	AD	→ Weight	NR	? Engagement ? Mood	8 weeks
Moore 2010 [65] USA	RCT	Nursing home and assisted living facility. Seated chair exercise with music	I = 43 C = 41	Yes	Various	Various	NR	↑ Quantity of food and fluid intake	→ Eating ability	3 weeks
Rolland 2007 [66] France	RCT	Nursing home. Exercise program including aerobic, strength, flexibility, and balance training, plus walking	I = 67 C = 67	Yes	Mild-severe	AD	→ Weight → MNA	NR	↑ Functional status	12 months

For abbreviations see below Table 2

**Table 6:** Summary of characteristics and results of four included multicomponent interventions, reported in four studies (for further detail see Additional file 2)

Study	Design	Setting, intervention type	No. of participants	Dementia diagnosed	Dementia stage	Dementia type	Effects on nutrition and/or hydration status	Effects on food, drink or nutrient intake	Quality effect (including QoL or meaningful activity) and other outcomes	Duration
Beck 2010 [67, 96] Denmark	RCT	Nursing home. Multicomponent (nutrition, exercise & oral care)	I = 62 C = 59	NR	NR	NR	↑ BMI ↑ Weight	→ E intake ↑ Protein intake	→ Mortality → Cognitive status → Functional status	11 weeks
Boffelli 2004 [34] Italy	BA	Dementia unit. Diet & environment modification, feeding assistance and supplements	29	Yes	Severe	various	→ BMI → weight ↑ albumin → Malnourished	NR	NR	18 months
Keller 2003 [35, 97] Canada	CCT	LTC facilities. Individualised food service, food modification, education and dietetic time	I = 33 C = 49	Yes	NR	AD & others	↑ Weight	NR	↑ Dietetic time → Hospital days → Mortality → Infections	30 months
Simmons 2001 [36, 98] USA	CCT	Nursing Homes. Staff assistance, prompting, food/drink service and exercise	I = 48 C = 15	NR	NR	NR	→ Serum osmolality, → BUN: creatinine ratio	→ Food & fluid intake	NR	32 weeks

For abbreviations see below Table 2

The diagnosis of dementia was stated in 45 interventions, while in the remainder dementia or MCI was assumed from cognitive scores or setting. Dementia staging was possible in 36 interventions (four mild to moderate, two moderate, six moderate to severe, 11 severe, 12 mixed, or mild to severe, and one MCI). Thirty-one interventions reported dementia type, of which 14 were Alzheimer’s Disease (AD), 10 were AD plus other types, and seven various or mixed types of dementia.

Indirect interventions were broadly grouped into; 17 dining environment/food service interventions, 15 interventions providing education or training, 12 behavioural interventions, eight exercise-type interventions and four multicomponent interventions (Fig. 1).

### Dining environment and food service

Seventeen interventions [19–33] investigated effects of changes to aspects of the dining environment or food service, and were reported in 15 studies, Table 2. Three multicomponent studies [34–36] also included an element of dining environment change, results are discussed with multicomponent studies. Three interventions were CCTs, [21, 22, 25] the remaining 14 had a pre-post design (BA, no RCTs). Interventions were assessed in over 450 participants, and intervention duration was from 5 days to 1 year. Interventions were primarily tested in North America (12 interventions), with three in Europe and two in New Zealand. All interventions took place in institutional settings (six dementia units, three nursing settings, six long-term care and two ‘other’ institutions). The variety of interventions and outcomes made meta-analysis (statistical pooling) unfeasible.

All interventions had a high risk of selection bias (none were RCTs) and were either at high or unclear risk of performance and detection biases (Additional file 2), none had a low risk of bias overall.

Two interventions assessed effects of home-like dining environment. Charras 2010 [21] (CCT) assessed mealtimes shared with staff in 18 residents with severe dementia while Altus 2002 [19] (BA) experimented with family style meals for five US females in a locked dementia unit. Charras reported increased weight (+5.64 kg, *p* < 0.024) after 3 months compared to the control group, and suggested (but did not measure) improved levels of autonomy (eating independently, helping with serving and clearing up), less wandering, longer meals, greater staff satisfaction, more and better interactions between residents as well as between residents and staff. Altus reported increased participation and communication during meals after 5 days compared to baseline but with no significance testing.

Two studies (Desai 2007 CCT, Shatenstein 2000 BA) [22, 31] compared the effect of bulk food service (cafeteria style with waitress service) to pre-plated or tray service in long term care Canadian residents with dementia, both reporting increased intakes of energy protein and carbohydrates, but no effect on weight or body mass index (BMI) over 3 or 10 weeks. Shatenstein found significantly decreased albumin status.

Three BA studies tested effects of music during mealtimes [30, 32, 33]. Thomas 2009 [32] played ‘familiar’ music at lunchtimes on alternate weeks to 12 US AD unit residents with moderate dementia. Wong 2008 [33] assessed ‘soothing’ mealtime music with 28 US inpatients with dementia during their period four (they introduced different interventions in each of four periods), and Ragenskog 1996 [30] tried three types (soothing music, tunes from the 1920’s and 30’s, and pop music) over dinner for 8–10 days for 20 Swedish nursing home residents with moderate or severe dementia. Pop music (but not other sorts of music) appeared to increase food intake and music generally improved several elements of psychological wellbeing in Ragneskog, [30] while ‘soothing’ music improved BMI but not mid-arm circumference in Wong, [33] with no clear effects of ‘familiar’ music on food intake, but reports of increased social engagement, remaining longer in dining area and response to the music in Thomas.

Improved dining room lighting and/or table setting contrast was tested in five BA interventions [20, 23, 26, 27]. There was no effect on weight, energy or fluid intake in response to different lighting and noise levels in 16 residents of US dementia units with AD, [27] but one of two lighting and contrast interventions (in 25 US long-term care residents with dementia, but not in 13 US residents of dementia units) reported improved intake, [20, 26] though not of functional status, weight or food quality. One of two studies comparing high and low contrast coloured tableware with white (in nine US men with AD, but not in the same 9 in a later intervention) improved fluid intake, but neither increased food intake [23].

Wong 2008 [33] (period 2, BA) found improved BMI (though not mid-arm circumference, with unclear effects on energy intake) after 12 weeks of using a glass-door fridge filled with snacks accessible at all times and earlier meals for those requiring more assistance or time in 40 in-patients with dementia.

Three interventions (Kenkman 2010 CCT, Perivolaris 2006 and Van Ort 1995, BA studies) [25, 28, 29] each tested the effect of a suite of dining environment changes, including offering more choice, less noise, fewer distractions, greater staff availability and a home-like environment, finding no clear effects on nutritional status, eating behaviour or enjoyment, but increased energy intake in Perivolaris [29] only. There were non-numeric suggestions of more leisurely and relaxed meals with less wandering in Perivolaris, and improved self-feeding behaviour in Van Ort.

One BA intervention [24] introduced an aquarium to the dining room of 70 US residents with severe dementia living in specialised units, reporting increased food intake after 8 weeks and suggesting weight gain (+2.2 lb, not statistically significant according to reviewers calculations).

### Education/training

Fifteen interventions assessed education or training [19, 29, 37–49] for people with dementia and/or their formal or informal care-givers, and were reported in 15 studies. In 12, education/training was the only intervention, in three it was one of two components [19, 39, 40], see Table 3 (one [29] was part of a multicomponent intervention). Six were RCTs, four CCTs and five BA including over 2100 people with dementia, with intervention durations from 5 days to 33 months. All interventions except Suominen 2013 [48, 50] were at a high or unclear risk of selection bias, all had high or unclear risk of performance bias and only Kwok 2012 [41] was at low risk of detection bias (see Additional file 2). No interventions were at low risk of bias overall.

Nine interventions investigated training of formal care-givers (staff), [19, 29, 38–40, 42, 43, 48, 49] two informal care-givers, [37, 45] and four trained people with dementia plus their formal or informal care-givers [41, 44, 46, 48]. The nine staff education interventions (one RCT, three CCTs, five BAs) varied from 3 h of web-based training [38] to 38 h of dementia-specific integrity training (lectures and discussion groups) followed by 3 months intensive support [42]. Only Mamhidir 2007 [42] (CCT), which provided the most intensive staff training and support reported positive effects of education on nutritional status, finding improved weight (+4.6 kg, *p* < 0.01) after 4 months, and the suggestion of improvements in mealtime environment and routines with increased contact between patients and staff. In Faxen-Irving 2002 [39] (CCT), where staff education was alongside oral nutritional supplements (ONS), weight gain occurred during supplementation but weight fell following withdrawal of supplement (*p* < 0.01) suggesting supplementation was more useful than staff education. Suominen 2007 [47] (BA) reported no effect on weight or BMI, but increased energy and protein intakes and self-reported staff confidence in nutritional assessment and modification, in 21 residents of Finnish dementia units following 6 months intensive education for nursing and catering staff. Staff training over 4 weeks in implementation of hydration management guidelines in Mentes 2003 [43] (BA) did not alter hydration status or fluid intake of eight US nursing home residents with dementia. The addition of staff training to family-style meals in Altus 2002 [19] (BA) appeared to increase mealtime participation and appropriate communication by people with dementia, but no variance or p-values were reported.

Riviere 2001 [45] (CCT) assessed effects of education (providing nine sessions on nutrition and preventing weight loss over a year) for informal caregivers of Europeans with AD. The intervention improved weight (1.4 kg, *p* < 0.05) compared to usual care. Hanson 2010 [37] used decision aid training for the US surrogates of people with severe dementia and feeding problems in an RCT. After 9 months there was a significant decrease in the percentage of patients with weight loss compared to control, but knowledge, decisional conflict and certainty were only assessed for the intervention group.

Four RCTs investigated effects of education of both people with dementia and their caregivers. Kwok 2012 [41] recruited formal care staff and residents with MCI from 14 Hong Kong hostels. Intervention participants received 1 h-long talk and 33 months of 3-weekly (later 6-weekly) support group sessions promoting “brain preservation diets” (more fruit, vegetables and fish, reduced salt), with training and support for staff, but despite the large sample (429 participants) and long duration, there was no effect on nutritional status or intake, except for a smaller fall in fish intake compared to control, with no effect on cognition. Three RCTs trained people with dementia and their informal caregivers [44, 46, 48]. NutriAlz [46] included over 900 people with mild to moderate dementia and their care-givers, providing 12 months nutrition education but reported no significant effect on weight, BMI, eating behaviour, caregiver burden or cognitive status, though an improvement in nutritional risk scores. Pivi 2011 [44] with over 50 patients and 6 months nutritional education intervention provided unclear statistics suggesting no effect on BMI, weight, arm circumference, triceps skinfold or serum albumin compared to control. Suominen 2013 [48] assessed effects of tailored nutritional training to people with AD and their spouses, preliminary results showing no effect on weight, but significantly increased protein intake and some suggestion of quality of life improvement.

### Behavioural interventions

Twelve behavioural interventions were assessed, [28, 51–56] in six RCTs, three CCTs and three BAs, reported in ten studies, Table 4. Studies reported on 347 people with dementia with study durations from 5 days to 6 months. All the studies were in institutional settings, six in North America and six in Asia. Risk of bias is represented in Additional file 2: ten of 12 interventions had unclear or high risk of selection bias, all had high or unclear risk of performance bias but eight of 12 had a low risk of detection bias. None were at low risk of bias overall.

Four interventions assessed mealtime staff prompting with cues, conditioning, reinforcement or gentle touch, [28, 51–53] with durations from 5 days to 2 weeks, too short to show nutritional status change (none were seen). Coyne 1988, [52] in a US RCT compared effects of directed verbal prompts and positive reinforcement with usual care in 24 nursing home residents with severe dementia. After 2 weeks, eating independence was significantly improved for solid foods, but not liquid foods, and eating frequency of solid and liquid foods was not altered. Eaton 1986 [53] (RCT) evaluated encouragement of eating through gentle touch in 42 self-feeding skilled facility residents with chronic organic brain syndrome. Five days gentle touch significantly increased energy and protein intake compared to verbal encouragement alone (*p* < 0.05). Van Ort 1995 [28] investigated effects of systematic prompting, cueing and behavioural guidance, delivered as a 4-week crossover RCT in seven residents with severe dementia in a secure unit of a US geriatric centre. The behavioural intervention resulted in longer mealtimes, and self-feeding increased. Weight change was not reported as resulting from either intervention. Beattie 2004 [51] (BA) assessed a mealtime intervention (conditioning over 20 min each evening meal of reinforcement and hand pressure on shoulder and “grabbing” dominant arm and re-seating if resident left table) in three US nursing home residents with severe AD and low food intake thought due to leaving the table early. There were no significant effects on weight but proportion of food eaten increased almost significantly in two of three participants (by 22 % and 35 %, *p* = 0.05) without increased fluid intake.

Five Taiwanese interventions assessed spaced retrieval or Montessori based activities [55, 56]. In spaced retrieval therapy several functional targets are selected, then one target is focused on until maintenance level is achieved, when another target is added. Montessori activities are free activities within a prepared or structured environment. The results were not conclusive nor consistent, except that all appeared to improve eating ability. Lin 2011 [56] (RCT) reported no effect of Montessori activities on BMI or mini-nutritional assessment (MNA), but improved functional and eating ability, self-feeding frequency and unaltered eating time) while Lin 2010 [55] (RCT) reported no effects of either 8 weeks of spaced retrieval or Montessori activities on weight or BMI (but improved nutritional risk with spaced retrieval only and improved eating difficulty for both) in people with dementia and eating difficulties. Wu 2013 [57] was not randomised (CCT) and found significant improvements in BMI and MNA (reduced nutritional risk) for fixed and individualised spaced retrieval combined with Montessori-type activities for people living in dementia units over 6 months.

McHugh 2012 [58] (RCT) found no effect of 3 weeks pre-lunch singing and music therapy sessions (11 familiar slow to medium tempo songs) on nutritional intake or participation in eight US adults with dementia in a memory support unit, while Huang 2009 [54] (BA) reported significantly greater feelings of happiness after 8 weekly sessions of reminiscence cooking in 12 Taiwanese nursing home residents with dementia but did not report on the nutritional status or intake.

Santo Pietro 1998 [59] (CCT) investigated the effect of a facilitated breakfast club where US nursing home residents with mid-stage dementia prepared, ate, cleared and conversed. After 12 weeks the intervention group had higher interest and involvement scores and better procedural memory with reports of participants remembering each-others names, increased use of humour, response to non-verbal cues, singing and empathic behaviour, with reduced wandering and distractibility (unclear effects on functional and cognitive status).

### Exercise interventions

We included eight exercise interventions, [60–66], reported in seven studies, Table 5, and exercise was also part of two multicomponent interventions [36, 67]. Six interventions were RCTs, [60, 62, 63, 65, 66] 2 BA, [61, 64] reporting on ~700 participants lasting from 3 weeks to 12 months. Three interventions were tested in North America, four in Europe and one in Asia, and settings included institutions (three in nursing homes, one long-term care facility (LTC) three in a mixture or other institutional settings) and daycare (one intervention). Four interventions, reported in 3 studies, were judged at low risk of selection bias (Dechamps 2010 [62], Rolland 2007 [66], FOPANU [60]), but all were at high or unclear risk of performance bias. Three interventions were at low risk of detection bias and six low risk of attrition bias, see Additional file 2. Two RCTs were at low risk of bias overall [60, 66].

Exercise interventions did not appear to improve nutritional status in any study, but there were indications of changes in strength and functional status in some. FOPANU [60] tested the effect of 3 months high intensity exercise (with or without timed protein-enriched supplement) vs. sitting activity (with or without supplement) in a 2×2 RCT on 191 functionally and cognitively impaired residents of nine Swedish residential care facilities. At 6 months, weight and intracellular water were lower in those with exercise training, while gait speed and lower limb strength were greater, mortality and balance unchanged. FICSIT [63] assessed the effect of 10 weeks of high intensity exercise vs placebo activities (both with or without ONS in a 2×2 RCT) in 100 mostly cognitively impaired institutionalised US elders, finding no effects on weight, thigh muscle area, body potassium, physical activity or mortality but increased energy intake, muscle strength and mobility. Dechamps 2010 [62] (RCT) compared adapted tai-chi and cognition action (light to moderate intensity seated exercises) to usual care in 160 French nursing home residents with cognitive impairment or dementia. After 6 months, eating independence was better maintained in the two exercise intervention groups compared to control, though cognitive function did not differ. Moore 2010 [65] assessed physical activity to familiar music compared to usual care in a US RCT in institutionalised older adults with dementia, finding increased food and drink intakes, but no effect on eating ability. Rolland 2007 [66] (RCT) investigated the effectiveness of an exercise program, which included aerobic, strength, flexibility, and balance training, plus walking on 134 French nursing home residents with mild to severe AD, finding no effects on weight or nutritional risk, but slower decline in functional status compared to usual care.

Chang 2011 [61] (BA) assessed effects of stretching, walking and weight bearing in 29 people with dementia in Taiwanese day care, finding a significant increase in functional status, but not eating function. Heyn 2003 [64] (BA), assessed effects of an 8 week multisensory exercise (focussed on attention, flexibility, aerobic exercise, strength training, relaxation & breathing techniques) in 13 US residents of a memory care residence with severe AD, finding no weight effect but promising though unclear effects on engagement and mood.

### Multicomponent interventions

Of four included multicomponent interventions [34–36, 67], reported in four studies, one was an RCT, [67] two CCTs [35, 36] and one BA, reporting on 295 people with dementia with durations of 11 weeks to 30 months, Table 6. All the studies were in institutional settings, two in North America and two in Europe. All multicomponent interventions were at high risk of selection and performance biases, one was at low risk of detection bias and one at low risk of attrition bias (Additional file 2). None were at low risk of bias overall.

Each intervention used different components. Beck 2010 [67] implemented nutrition (chocolate plus homemade supplements, gratin diet for people with swallowing difficulties), exercise (individualised sessions twice weekly), and twice weekly oral hygiene for 11 weeks in an RCT, finding increased weight, BMI and protein intake with no change to energy intake, mortality, cognitive or functional status. Keller 2003 [35] used an enhanced menu and dietetic time, increased nutritional awareness and communication (CCT) for 9 months and increased participant weight and dietetic time, without altering hospital stay duration, infections or mortality. Simmons 2001 [36] (CCT) prompted US nursing home residents to drink and exercise, and offered them assistance with getting to the toilet and/or checked for incontinence every 2 h for 8 h/day for the first 16 weeks, rising to 10 h/day for the next 16 weeks, plus increased drinks choice for the final 7 weeks. They found no effects on serum osmolality, BUN:creatinine ratio or food and fluid intake at or between meals. Boffelli 2004 [34] (BA) implemented an 18 month nutritional program for malnourished people with dementia that included modification of dietary composition, quality and consistency (modified on preference, swallowing ability, dental status), increased feeding time and assistance, enhanced dining environment and ONS prescribed for low intake, finding improved albumin but unchanged weight or BMI.

## Discussion

This review systematically assessed the effectiveness of 56 indirect interventions including: environmental, educational, behavioural, exercise and multicomponent interventions aiming to improve, maintain or facilitate oral food or fluid intake in adults with dementia of any type or stage and in any setting. While almost all included studies were set in institutions, they varied enormously in the type, intensity and duration of interventions, as well as recorded outcomes. Generally study validity was low – only 19 (34 %) of interventions were assessed in RCTs, of these, five interventions were at low risk of selection bias, none were clearly at low risk of performance bias, and 13 were at low risk of detection bias. We considered two interventions to be low risk of bias overall, [60, 66] both investigated exercise interventions. This high risk of bias alongside small numbers of included participants assessing many interventions means that no interventions can be clearly ruled in or ruled out as effective. We may be seeing exaggerated effect sizes where we see significant effects, but important and effective interventions may be underpowered (and of too short duration) to provide statistically significant effect sizes, so we may be missing important interventions. It is likely that it is not just what people eat and drink that is important for their nutritional wellbeing, engagement and quality of life, but also how and where they eat and drink, the atmosphere, physical and social support offered, the understanding of formal and informal care-givers, support for using the toilet, and levels of physical activity enjoyed – but for people with dementia any proof of this has yet to be published [68]. However, promising interventions, which warrant early reassessment in high quality and well powered RCTs are shown in Table 7.

**Table 7:** Promising interventions that are presently unproven, but that warrant early reassessment in high quality and well powered RCTs^a^

Aim	Potential interventions (presently unproven) which warrant early reassessment
Increase weight and/or BMI	o Eating meals with care-givers eating alongside (Charras) o Soothing mealtime music (Wong 2008) o Glass-door fridge with constantly accessible snacks and additional time for meals (Wong) o Extensive staff education and support (Mamhidir) – though smaller amounts of support are not so promising o Education and support for informal care-givers of people with dementia (Riviere and Hanson) o Spaced retrieval and Montessori activities (Wu 2013) o Multicomponent intervention including chocolate supplements, gratin diet, exercise and oral hygiene twice weekly (Beck) o Multicomponent intervention including enhanced menu, more dietetic time, increased nutritional awareness and communication (Keller)
Improve hydration	o No very encouraging interventions found
Supporting meaningful engagement with food and/or drink	o Eating with care-givers (Charras) o Family style meals for people with dementia, enhanced further by staff training (Altus) o Extensive staff education and support (Mamhidir) o Facilitated breakfast club with supported involvement in preparing, conversing, eating and clearing up (San Pietro) o Multisensory exercise (focussed on attention, flexibility, aerobic exercise, strength training, relaxation & breathing techniques, Hayn)
Quality of life	o Reminiscence cooking sessions (Huang 2009) o Appropriate, particularly familiar, music during meals (Thomas, Ragneskog) o Tailored nutritional training to people with AD and their spouses (Suominen 2013)
Supporting eating independence	o Directed verbal prompts and positive reinforcement, systematic prompting, cueing and behavioural guidance (Coyne, Van Ort) o Spaced retrieval (Lin 2010) o Montessori activities (Lin 2010, 2011) o Adapted tai-chi (Dechamps 2010) o Cognition action (light to moderate intensity seated exercises, Dechamps 2010)
Quantity, quality or adequacy of food or fluid intake	o Bulk food service (rather than pre-plated or tray service, Desai, Shatenstein) o Pop music during meals (Ragneskog) o Some lighting and contrast interventions to improve visual cues (Brush 2002, Dunne) o Encouragement of eating through gentle touch (Eaton) o Physical activity to familiar music (Moore) o High intensity exercise (FICSIT)

^a^if you or someone you care for is experiencing difficulties with eating or drinking ALWAYS discuss these eating and drinking problems with your/their doctor, and ask to be referred to a dietitian and/or Speech and Language Therapist

Research gaps include a shortage of potentially useful interventions to support drinking and healthy fluid intake, and shortages of research supporting people with dementia living in the community rather than in institutions. Fluid intake appears lower in older adults, the very group most at risk of dementia, [69] which may endanger renal function, endocrine and cardiovascular function and is associated with increased risks of mortality and disability [70–74]. People with dementia and their care-givers appear rarely to be involved in developing appropriate interventions – the support needs and preferences of people with dementia, their informal and formal care-givers need to inform the research and policy agendas.

Strengths of the review include a protocol registered on Prospero, [8] involvement of service users and stakeholders, an experienced review team, an exhaustive literature search, duplicated assessment of inclusion, duplicated data extraction, extensive assessment of study validity, and detailed tables of information on the included studies. Weaknesses of the review include our inability to statistically pool outcome data (in meta-analyses) as interventions and outcomes were not similar enough, and the small size and low validity of the included research, which mean that we are not able to label specific interventions as either effective or ineffective. However, the review does provide a list of potentially useful interventions that people with dementia and their care-givers may like to try, to deal with specific problems, and which researchers may use to prioritise future, high quality, research.

This review, along with its sister review on direct interventions, [6] is the first comprehensive systematic review of interventions to support eating and/or drinking in people with dementia and mild cognitive impairment. Previous systematic reviews have concentrated on nutritional interventions to maintain cognitive function, [75, 76] explored the effectiveness of mealtime interventions without addressing between-meal interventions, were not specific for people with dementia or did not include nutritional status or quality as outcomes [11–13, 17]. Other systematic reviews addressed effectiveness of various indirect interventions on people with dementia but did not assess impact on nutritional or hydration status [14–16, 77].

## Conclusions

Malnutrition is associated with poor quality of life, [78] and problems with malnutrition, dehydration, poor eating and drinking are common in people with cognitive impairment, so understanding how to help continue eating and drinking well is very important in supporting health and quality of life of people with dementia. However, we found that studies were small and there were no clearly effective, or clearly ineffective, interventions. Promising interventions included: eating meals with care-givers, family style meals, soothing mealtime music, constantly accessible snacks and longer mealtimes, education and support for formal and informal care-givers, spaced retrieval and Montessori activities, facilitated breakfast clubs, multisensory exercise and multicomponent interventions. High quality research is needed to build on existing research, summarised in this review, to help understand what types of interventions are effective in supporting adults with MCI or dementia to eat and drink well, and to remain actively engaged with food and drink.

### Ethics approval and consent to participate

Not applicable.

### Consent for publication

Not applicable.

### Availability of data and materials

Our data files are found in Additional file 2, and the references to each included study in the main paper.

## Additional files

Additional file 1: Full systematic review methodology for EDWINA (Eating and Drinking Well IN dementiA) systematic review. (DOCX 30 kb) Additional file 2: Tables of characteristics of included studies and risk of bias summaries. (DOCX 216 kb)

## Abbreviations

AD: Alzheimer’s disease
BA: before/after study
BUN: blood urea nitrogen
CCT: non-randomised controlled trial
EDWINA: Eating and Drinking Well IN dementiA study
MCI: mild cognitive impairment
MMSE: mini-mental state examination
MNA: mini-nutritional assessment
ONS: oral nutrition supplements
RCT: randomised controlled trial
