# Supplementary material for: Effectiveness of interventions to indirectly support food and drink intake in people with dementia: Eating and Drinking Well IN dementiA (EDWINA) systematic review
Source: BMC Geriatr. 2016 May 4;16:89. doi: 10.1186/s12877-016-0256-8 (PMC4855348; doi:10.1186/s12877-016-0256-8)
Supplement: Additional file 1: — Full systematic review methodology for EDWINA (Eating and Drinking Well IN dementiA) systematic review. (DOCX 30 kb) [file 12877_2016_256_MOESM1_ESM.docx]

**Additional file 1. Full systematic review methodology for EDWINA (Eating and Drinking Well IN dementiA) systematic review**

The protocol for this systematic review was developed collaboratively by the review team, which included subject experts, and members of the public and is published on Prospero^1^. The review team included Lee Hooper (University of East Anglia, UEA), Chris Fox (Norfolk and Suffolk NHS Foundation Trust, and UEA), Asmaa Abdelhamid (UEA), Diane K Bunn (UEA), Maddie Copley (Age UK Norfolk), Vicky Cowap (NorseCare), Angela Dickinson (University of Hertfordshire), Amanda Howe (UEA), Anne Killett (UEA), Fiona Poland (UEA), John F Potter (Norfolk and Norwich University Hospital NHS Trust, NNUH)), Kate Richardson (NNUH) and David Smithard (King’s College Hospital NHS Foundation Trust). The team included expertise in systematic review methodology (LH, AA, DKB), dementia and cognitive impairment (CF, MC), care home provision (VC), research with older people (LH, CF, AD, AK, FP, JFP), understanding the needs of older people (MC, VC, AK, AD), working with patient and public groups in a research context (AD, AK, FP, LH, DKB), hospital medicine (CF, JFP, KR, DS), primary care (AH, CF, AA), swallowing problems (KR, DS), occupational therapy (AK), nutrition and dietetics (LH), and hydration (DKB, LH), as well as lay stakeholders (MC, VC). The team also worked with two patient and public involvement in research groups to develop further specific questions for the review to address. The review was conducted in accordance with Cochrane Collaboration’s guidelines, and reported in accordance with PRISMA guidance.

**Criteria for inclusion:**

We included intervention studies that included at least three adult participants with any type and stage of dementia (mild to severe) or mild cognitive impairment (MCI), in any setting, utilising formal or informal care or no care at all. Where dementia status was not clearly stated, dementia or cognitive impairment was assumed if the sum of the mean Mini Mental scale score plus one standard deviation was ≤26. Studies that included dementia patients among other groups were considered where results for dementia patients could be separated or those with dementia constituted at least 75% of participants. Studies had to have a control group and minimum duration of five consecutive days. Study designs included: randomised control trials (RCTs), cluster randomised trials (cRCTs), quasi-experimental and pre-post studies (CCTs). Cross-over RCTs or CCTs were included, but only contributed to short term outcomes such as food or drink intake.

Interventions had to aim to increase, facilitate or maintain oral food and/or drink intake, nutrition or hydration status, and have a direct or indirect effect on food and fluid intake. Direct interventions aimed to modify food and drink, provide food-based supplements, assist with eating or drinking or manage swallowing problems, whilst indirect interventions altered the dining environment, altered food service, provided education or training of people with dementia or their carers, behavioural interventions, exercise, or multi-component interventions. A question was raised while conducting the review, on whether interventions that focussed on improving social contact of people with dementia in the context of food and drink were successful in supporting review outcomes. This led to the addition of a new category to the direct intervention groups. Pharmacological interventions (such as pharmacological appetite stimulants and non-food supplements) were excluded. The review has been published as two papers, one focussing on direct interventions, one on indirect interventions.

Included studies had to assess some measure of nutrition or hydration status, quantity, quality or adequacy of food or fluid intake (including ability to eat independently, and ability to swallow without aspirating), meaningful activity or enjoyment of food or drink (activity around food or drink that is personally fulfilling, that people enjoy, look forward to or find important). Where included studies also assessed quality of life, functional or cognitive status, views or attitudes, cost effectiveness or resource use, mortality, or health outcomes these were also recorded.

**Search strategy:**

We searched the following databases combining text and indexing terms, truncation and Boolean operators for relevant studies; MEDLINE, EMBASE, CINAHL, PsychInfo, the Cochrane Library (including Cochrane Central Register of Controlled Trials (CENTRAL), Cochrane Database of Systematic Reviews, Database of Abstracts of Reviews of Effects, Health Technology Assessment, NHS Economic Evaluation Database), the meta-register of controlled trials (a trial register that includes the International Standard Randomised Controlled Trial Number Register, ISRCTN, and the NIH Clinical Trials Register), ALOIS (Cochrane Dementia and Cognitive Improvement Group comprehensive register of dementia trials) and Dissertation and Thesis abstracts, the International Alzheimer's Disease Research Portfolio (IADRP). Bibliographies of included studies and lists of included and excluded studies from relevant existing systematic reviews were checked. The search was not limited by language or time period. The full Medline search strategy is found in the published protocol ^1^. It was tested against a set of studies known to fit the inclusion criteria, to ensure the search strategy was working well, then modified accordingly. The MEDLINE strategy was adapted for the other databases.

**Study selection and data collection**

Titles and abstracts were assessed against inclusion criteria by two reviewers independently and full text copies of all papers obtained if either reviewer considered it potentially eligible. An inclusion form was used to assess full text studies for inclusion or exclusion. Differences were resolved by discussion and when necessary were arbitrated by a third reviewer. Multiple publications from the same study were grouped.

Data and validity characteristics were extracted in duplicate from included studies using a form designed for this purpose, and included bibliographic information (study authors, year and country of publication, details of multiple publications), study design, details of study participants (inclusion criteria, number, age, sex, type of dementia, diagnostic criteria, stage of dementia, setting), interventions (description of intervention, duration, details of comparator), and outcomes. For each study we extracted numbers of events and numbers of participants in each arm for categorical data. For continuous data we extracted change data and the standard deviation of the change, and number of participants for each arm. Where change data are not provided then we used end data (outcome data at the end of the intervention).

Differences between reviewers were resolved through discussion and a third reviewer arbitrated when needed. We attempted to contact researchers where inclusion or exclusion could not be decided based on available data.

**Study validity**

The methodological validity of each included study, and its risk of bias, was assessed using Cochrane risk of bias tool ^2, 3^. In addition to the generic criteria we assessed the validity of methods of diagnosis of dementia, of outcome measures, funding and baseline comparability between groups. We considered a study at low risk of bias where it had a low risk of selection bias (was randomised and had appropriate allocation concealment) and a low risk of detection bias (with blinding of outcome assessment).

**Data synthesis**

The search and inclusion processes were presented using a flow chart. Characteristics and quality of included studies, and details of studies assessed in full text but excluded, were tabulated. Summary tables indicated which studies showed an increase or decrease in an outcome where a statistically significant difference between intervention and control were demonstrated.

In the main analysis, studies were grouped by type of intervention then by study design for tables and narrative synthesis. Where a study tested two different interventions simultaneously on the same group, the study was included in both groups following discussion of the single interventions. Studies with at least three intervention elements (not including the social element) were considered and grouped together as multicomponent interventions. Meta-analysis was conducted where studies were suitably comparable. Results were pooled in random-effects meta-analysis using Review Manager (RevMan 5.3) software. Heterogeneity was quantified using I^2^ ^2^. The main analysis included all RCTs for each type of intervention with relevant outcomes (non-RCTs were compared narratively to meta-analysis results). As interventions are most useful if they have a lasting effect on nutritional status subgrouping was by duration of intervention (up to 12 weeks, >12 weeks to 6 months, >6 months.

We intended to assess publication bias using funnel plots, but there were insufficient studies pooled to carry this out.

Secondary analyses used subgrouping to address the questions developed by our public involvement, and also addressed a question that was raised during the conduct of the review, on whether interventions that specifically focussed on improving social contact of people with dementia in the context of food and drink were successful in supporting relevant review outcomes.

**Specific questions** to be addressed by the review were formulated by the research team following consultations with members of two patient and public involvement groups (the Public & Patient Involvement in Research, PPIRes, from Norfolk and Suffolk and the Public Involvement in Research Group, PIRG, from the University of Hertfordshire). The groups, and their members, were asked to comment on the protocol and let us know what questions they would like the review to address. The questions to be addressed by the review included:

1. What are the most effective ways to encourage people with dementia to eat, drink and maintain nutritional intake?
2. For people with mild dementia, what interventions can help to maintain or improve food intake or nutritional status?
3. For people with mild dementia, what interventions can help to maintain or improve fluid intake or hydration status?

- (repeat questions 2 & 3 for mild cognitive impairment, moderate dementia and severe dementia)

1. For people with dementia living in their own homes with a carer, what interventions can help to maintain or improve food intake or nutritional status?
2. For people with dementia living in their own homes with a carer, what interventions can help to maintain or improve fluid intake or hydration status?

- (repeat questions 4 and 5 for those living at home with a part time or no carer, for those living in residential care, for those in hospital)

1. For people with Alzheimer’s dementia, what interventions can help to maintain or improve food intake or nutritional status?
2. For people with Alzheimer’s dementia, what interventions can help to maintain or improve fluid intake or hydration status?

- (repeat questions 6 and 7 for those with vascular dementia, Dementia with Lewy bodies, mild cognitive impairment, other types of dementia, and mixed populations)

1. For people with dementia, what interventions aimed at improving or maintaining food and/or fluid intake, nutrition or hydration status, support meaningful activity (activity around food or drink that is personally fulfilling, that people enjoy, look forward to or find important)?
2. For people with dementia, are there any interventions that; worsen food or fluid intake, worsen enjoyment or quality of life, or worsen meaningful activity or social inclusion?
3. Do individualised interventions appear more effective than those that are not individualised, in helping people with dementia to maintain or improve food and/or drink intake, nutrition or hydration status (or related outcomes)?
4. Do interventions to assess swallowing (and where necessary treat swallowing problems) have any effect on food or drink intake, nutrition or hydration status (or related outcomes)?
5. Do interventions to improve oral hygiene have any effect on food or drink intake, nutrition or hydration status (or related outcomes)?”
6. For people with dementia living in the community, does type of carer providing the intervention affect the outcomes (e.g. close relative vs paid carer, full time vs occasional carer)?
7. For people with dementia, does emotional closeness of the carer (e.g. close relative vs paid carer) affect the outcomes?
8. Are there any interventions that are particularly effective in helping people with dementia to maintain or improve food and/or drink intake, nutrition or hydration status (or related outcomes) during periods of acute illness?

References

(1) Abdelhamid A, Bunn D, Dickinson A et al. Effectiveness of interventions to improve, maintain or faciltate oral food and/or drink intake in people with dementia. *PROSPERO* 2014;CRD42014007611:<http://www.crd.york.ac.uk/PROSPERO/display_record.asp?ID=CRD42014007611> .

(2) *Cochrane Handbook for Systematic Reviews of Interventions Version 5.1.0 [updated March 2011]*. Oxford: The Cochrane Collaboration (Available from [www.cochrane-handbook.org.);](http://www.cochrane-handbook.org.);) 2011.

(3) Higgins JP, Altman DG, Gotzsche PC et al. The Cochrane Collaboration's tool for assessing risk of bias in randomised trials. *British Medical Journal* 2011;343:d5928.
