# Supplementary material for: Effectiveness of interventions to indirectly support food and drink intake in people with dementia: Eating and Drinking Well IN dementiA (EDWINA) systematic review
Source: BMC Geriatr. 2016 May 4;16:89. doi: 10.1186/s12877-016-0256-8 (PMC4855348; doi:10.1186/s12877-016-0256-8)
Supplement: Additional file 2: — Tables of characteristics of included studies and risk of bias summaries. (DOCX 216 kb) [file 12877_2016_256_MOESM2_ESM.docx]

**Additional file 2. Tables of characteristics of included studies and risk of bias summaries for Eating and Drinking Well IN dementiA: EDWINA systematic review” by Abdelhamid A et al.**

| **Supplemental item** |  | **Page** |
| --- | --- | --- |
|  |  |  |
| **Supplemental Table 1. Characteristics and results of interventions to the dining environment and/or food service (EDWINA)** |  | **2** |
| **Supplemental Table 2. Characteristics and results of education and training interventions (EDWINA)** |  | **13** |
| **Supplemental Table 3. Characteristics and results of behavioural interventions (EDWINA)** |  | **23** |
| **Supplemental Table 4. Characteristics and results of exercise interventions (EDWINA)** |  | **30** |
| **Supplemental Table 5. Characteristics and results of multicomponent interventions (EDWINA)** |  | **34** |
| **Supplemental Figure 1. Risk of bias summary for dining environment and food service interventions** |  | **38** |
| **Supplemental Figure 2. Risk of bias summary for educational intervention** |  | **40** |
| **Supplemental Figure 3. Risk of bias summary for 12 behavioural interventions** |  | **41** |
| **Supplemental Figure 4. Risk of bias summary for exercise interventions** |  | **42** |
| **Supplemental Figure 5. Risk of bias summary for multicomponent interventions** |  | **43** |
|  |  |  |

**Supplemental Table 1. Characteristics and results of interventions to the dining environment and/or food service (EDWINA)**

| **Study** | **Participants** | **Interventions** | **Outcomes*** | **1° outcomes*** | **2° outcomes*** | **3° outcomes*** |
| --- | --- | --- | --- | --- | --- | --- |
| **Altus 2002 (period 1)**  **USA**  **Setting:**  **Locked dementia unit**  **Aim:**  To examine if changing the mode of meal delivery to “family-style” would increase residents’ communication and participation in mealtime tasks. | **Participants**: Dementia unit residents  **N:** 5  **M/F:** 0/5  **Mean age (range)**: 80 (76-87)  **Nutritional status: NR**  **Cognitive status: mean** MMSE score (range) 8 (3-16)  **Dementia diagnosis:** physician diagnosis  **Dementia type:** AD or other dementia  **Dementia stage:** Moderate to severe  **Acute illness:** NR | **Design:** BA (time-series repeated measures)  **Int**: Family- style meals (with or without nursing assistant training)  **Details: ABAB’ design**  **Provided by:** health worker/care home staff  Details: **A** (baseline condition 10 d) **B** (family style meals 5 d) **A** (repeat 5d) **B’** (family style + assistant training 5d)  **Cont**: pre-plated meals  **Duration of int/ follow up**: 2 d (10int d/25)  **Compliance:** NR  **Individualised: No**  **Ethics obtained: NR** | **1º outcomes**:  Meaningful activity (% of resident participation in mealtime, appropriate & inappropriate communication, frequency of praise by nurse)  **2° outcomes**:  Views or attitudes of participants  **3° outcomes** | **Resident participation in mealtime tasks: (% of participation)**  24% after int 1 vs 10% baseline 1  65% after int 2 vs 6% baseline 2  **Appropriate communication:** (mean % of intervals with appropriate communication)  10.6% after int 1 vs 5.5% baseline 1  17.9% after int 2 vs 3.8% baseline 2  **Praise statements:** (frequency of praise statements by CNA**)**  7.2/meal post int 1 vs 0.2/meal baseline 1  14.2/meal post int 2 vs 0/meal baseline 2 | **CNA’s satisfaction with level of participation:**  post int: 5 (very satisfied) vs pre int: 2 (somewhat satisfied) |  |
| **Brush 2002**  **USA**  **Setting**  **2 Long term care facilities (LTCF)**  **Aim:**  To examine the effect of improved lighting & table setting contrast on residents’ oral intake & behaviour during meals in both assisted living & long term care environment serving people with dementia. | **Participants**: LTCF dementia residents  **N:** 25 (11at facility 1&14 at facility 2)  **M/F:** 3/22  **Age**: all >70  **Nutritional status:** no details  **Cognitive status:** MDS (not specific) some details on decision making ability **Dementia diagnosis:** physician diagnosis  **Dementia type:** NR  **Dementia stage:** Moderate-severe  **Acute illness:** NR | **Design: BA**  **Int Type:**Environmental (lighting modification)  **Provided by: care home staff**  Details: Halogen lights and extra fluorescent bulbs were added to the dining room in both facilities to reduce the ratios between the lightest and darkest parts. Also Navy blue liners were added under the plates to increase contrast at the table setting. Tables at one facility were covered in green non-glare cloths (however, changes didn’t increase lighting to the recommended levels)  **Cont**: N/A  **Duration of int/ follow up**: 4wks  **Compliance: NR**  **Individualised: No**  **Ethics obtained: NR** | **1º outcomes**: None  **2º outcomes**: Quantity of food intake (Energy intake)  Quality or adequacy of food intake (Meal Assistance Screening Tool, MAST)  Measures of functional status (Communication Outcome Measure of Functional Independence, COMFI)  **3º outcomes** |  | **3d Calorie count, Mean (SD) (also available for individual patients)**  Facility 1 baseline: 3277(1651)  Facility 1 post-test: 4339 (1762)  **(Increase of 1062, P=0.14)**  Facility 2 baseline: 3571 (936)  Facility 2 post-test: 4476 (853)  (increase of 905, P=0.007) (combined: Pre 3442(1278), Post 4416 (1301) P=0.008)  **Total COMFI score (of 0-100, higher score= better functioning), Mean (SD)**  Facility 1 baseline: 54 (20)  Facility 1 post-test:72 (11)  (P=0.018)  Facility 2 baseline: 48 (22)  Facility 2 post-test:61 (17)  (P=0.115)  **Total MAST score (higher score= severe problems), Mean (SD)**  Facility 1 baseline:10.7 (8)  Facility 1 post-test:10.8 (5) not sig  Facility 2 baseline: 6.2(3)  Facility 2 post-test:4.9 (3) not sig |  |
| **Charras 2010**  **France**  **Setting**  **2 Dementia units in nursing homes**  **Aim:**  To study the impact of changed mealtime experience for people with AD | **Participants**: AD patients from 2 special units  **N:** 18 (8 int, 10 cont)  **M/F:** Not reported  **Mean age (SD)**:85.2 (6.5)  **Nutritional status:** only weight reported  **Cognitive status:** mean MMSE score (SD) 7.5 (5.6) **Dementia diagnosis:** diagnosed (no details)  **Dementia type:** AD  **Dementia stage:** Severe  **Acute illness:** NR | **Design: cluster CCT**  **Int Type:**Dining environment (shared mealtime meals)  **Provided by: Nursing home staff**  Details: Shared lunchtime meals between staff & residents with individual help available when necessary. 2 large tables of 8 or 9 patients and staff. 4 courses offered.  **Cont**: Usual care  There was a 12 session training programme + environmental design int to increase orientation & wellbeing were implemented in both int and cont units.  **Duration of int/ follow up**: 3mo  **Compliance: NR**  **Individualised: No**  **Ethics obtained: NR** | **1º outcomes**:  Nutritional status (Weight)  Meaningful activity (autonomy, quality of interaction)  **2º outcomes**:  Views or attitudes of carers/ staff  **3º outcomes** | **Body weight, kg, mean (SD)**  Int: Baseline 51.3 (8.7), post int 54.6 (9)  Cont: baseline 63 (12.6), post int 60.8 (12.4)  int +3.4 kg vs cont -2.2 kg  P<0.0244 | **Meaningful activity & views: (qualitative data)**  **Reported by observations**  **Autonomy:**  Some trying to eat independently  Residents serving themselves and helping in clearing up  **Quality of interactions:**  Increase of resident-resident & staff-resident interaction  Increase of staff awareness of residents’ likes, dislikes and biography  Residents helpful to each other and increase talking.  **Attitudes:**  Less wandering by residents  More satisfaction and effort by staff  **Food quality:**  Increased as staff reported back  **Timing:**  Meals took 1-1.5 hrs presenting a challenge |  |
| **Desai 2007**  **Canada**  **Setting**  **Long term care**  **Aim:**  To compare energy intakes in seniors with cognitive impairment in long term care receiving meals by bulk vs traditional tray delivery systems and determine subject characteristics that identify responsiveness to type of food service provided. | **Participants**: AD independent patients of 2 care facilities  **N:** 48 (22 int., 26 cont)  **M/F:** NR  **Mean age (SD)**:88.8 (4.2) int, 86.2 (7.7) cont  **Nutritional status: BMI, mean (SD)** int, 23.8 (3.9), cont 21.9 (3.1)  **Cognitive status: LPRS (London psychogeriatric rating scale- mental disorganization)**  Int. 33 (11.2), cont 33.1 (11.1)  **Dementia diagnosis:** standardised assessment  **Dementia type:** AD  **Dementia stage:** NR  **Acute illness:** No | **Design: CCT**  **Int Type:** Bulk/ waitress food service  **Provided by: Health workers?**  Details: food service was changed to waitress delivery. Patients shown all foods. Environment was changed from institutional appearance to more home-like setting.  **Cont**: Pre-plated tray food service  **Compliance: NR**  **Individualised: No**  **Duration of int/ follow up**: 21d  **Ethics obtained: Yes** | **1º outcomes**: Nutritional status (BMI)  **2º outcomes**:  Quantity of food intake (Energy intake, protein, fat, CHO)  **3º outcomes** | **BMI**  **No numbers reported at 21 d** but figure presents relationship between BMI & energy intake. Lowest BMI subjects were more sensitive to foodservice type. | **Total energy intake, 24 hrs, kcal, mean (SE)**  Mean difference between int vs cont at 3 wks +265(55)  P<0.01  **CHO intake, g**  Mean difference between int vs cont (SE) at 3 wks +47(8.6)  P<0.001  **Protein intake, g**  Mean difference between int vs cont (SE) at 3 wks +6(2.8)  P<0.05 |  |
| **Dunne, 2004**  **Studies 1&2**  **USA**  **Setting**  **Long-term care unit**  **Aim:**  To examine how contrast manipulations may affect food and liquid ingestion. | **Participants**: AD patients who eat independently  **N:** 9 (study 1); 9 (study 2, 6 completed)  **M/F:** all men  **Mean age (range)**: study 1: 82.7(72-89)  Study 2: 83.1(NR)  **Nutritional status: NR**  **Cognitive status:** MMSE, mean (range) Study 1 2.9 (0-8), study 2: 3.2 (NR) **Dementia diagnosis:** Formal diagnosis of probable AD  **Dementia type:** AD  **Dementia stage:** Severe  **Acute illness:** No | **Design: BA (interrupted time series)**  **Int Type:** food service/ environment  **Provided by:** care home staff  Details:  **Study 1**: 10d high contrast red tableware (red plates, cups, and flatware) at lunch and supper time.  **Study 2**: 10d each of  1. High contrast blue tableware  2. Low contrast red  3. Low contrast blue  **Cont**:  Study 1: 10d baseline conditions of white tableware and stainless steel flatware before int and 10d after. Study 2: 10d of standard white tableware separating the int periods.  **Duration of int/follow up**: study 1: 30 d (10 int d)  Study 2: 70 d (3 x 10 int d)  **Compliance:** NR  **Individualised: No**  **Ethics obtained: NR** | **1º outcomes**: None  **2º outcomes**:  Quantity of food intake  Quantity of fluid intake  **3º outcomes**  Mortality |  | **Study1 (high contrast red)**  **% increase in food intake, mean (SD)**  baseline 71.2% (26.8%) Post int. 86.7% ( 19.3%) p=0.16 calculated (p=0.001 presented in paper  **% increase in fluid intake, mean (SD)**  baseline 54.4% (36.6%) Post int. 87.7% ( 22.1%)  P=0.03 calculated  (P=0.001 presented in paper)  **Study 2:**  **1.High contrast**  **blue condition**: food intake, mean (SD)  Baseline 63% (29%); Int 78% (25%)  P=0.25 calculated  **liquid intake,**  Baseline 77% (34%); Int 92% (21%) P=0.26 calculated  **2.low-contrast red condition**, **food intake**,  Baseline 63% (29%); int 62% (29%)  P=0.95 calculated  **liquid intake**,  Baseline 88% (25%); int 88% (25%) P=1.0 calculated  **3.low contrast blue** condition, **food intake**, mean (SD)  Baseline 58% (28%); int 62% (30%)  P=0.82 calculated  **liquid intake**,  Baseline 88% (25%);  Int 90% (22%) P=0.88 calculated | **Mortality:**  3 died during study 2 |
| **Edwards 2013**  **USA**  **Setting**  **Specialised dementia units**  **Aim:**  To assess whether patients with dementia who observe aquariums increase the amount of food they consume and maintain body weight. | **Participants**: orally fed residents of 3 dementia units  **N:** 70  **M/F:** 18/52  **Mean age (SD)**: 82.2 (7.1)  **Nutritional status: NR**  **Cognitive status:** MMSE, mean (SD) 5.57 (5.9) range 0-20 **Dementia diagnosis:** diagnosed- no details  **Dementia type:** NR  **Dementia stage:** severe  **Acute illness:** NR | **Design: BA**  **Int Type:** Environment (aquarium)  **Provided by: care staff**  Details: Introduction of an aquarium into the common area where dining occurred. The Rolling sea aquarium was designed for long term care facilities with 30x20 inch viewing area at level visible from wheelchair or at table with a lighted background and 8 large colourful fish.  **Cont**: No aquarium  **Duration of int/follow up**: 8wks  **Compliance:** NR  **Individualised:** No  **Ethics obtained:** Yes | **1º outcomes**: Nutritional status (weight)  **2º outcomes**:  Quantity of food intake, Quantity of fluid intake  **3º outcomes** | **Weight:**  Baseline: 158.4 lb,  post int 160.6 lb. Mean change +2.2 pounds.  (reviewer calculated p=0.65 but paper reports sig t-test results, p<0.001)  8/70 (11%) lost weight mean=1.89 pounds (0.13-7.38 CI) | **Mealtime food intake (g)** calculated separately for solids and beverages and for each meal but presented as t-test results (not numerically) for each meal.  **Total food intake (including solids & beverages)** increased by mean 196.9g from baseline to post int (P<0.001) |  |
| **Kenkmann 2010**  **UK**  **Setting**  **Care homes**  **Aim:**  To assess the health, wellbeing & nutritional status of a population of older people living in UK residential care, and to assess the feasibility of measuring the effects of a change in provision of food and drink in this context. | **Participants**: Residents of 6 care homes in Norfolk  **N:** 105 allocated (int. 57 from 3 NHs, cont 48 from 3NHs) 63 analysed.  **M/F:** 31/74  **Mean age (SD)**: 86 (6.7) int, 88 (6.8) cont  **Nutritional status: BMI, mean (SD) int.** 25.8 (5.3), cont 24.7 (4.5)  **Cognitive status:** MMSE, mean (SD) int. 19(5.6), cont 17(6.2)  **Dementia diagnosis:** NR  **Dementia type:** NR  **Dementia stage:** NR  **Acute illness:** Yes | **Design: CCT**  **Int Type:** Dining environment & menu changes  **Provided by:** care staff  Details: Changes included; improved dining atmosphere, readily available snacks and drinks machines, increased food choice, extended restaurant hours and self-service snacks all the time.  The changes aimed to improve comfort during meals, increase the level of choice available at meal times, making eating with others a pleasurable and more sociable experience and encourage fading appetites. They were also intended to widen the availability of drinks and snacks (to visitors  as well as residents), encourage greater independence on  the part of residents in choosing and obtaining their own snacks, and generally reduce the feeling of institutionalisation  **Cont**: standard care (limited menu choice, fixed meal, drinks and snack times, no visitors joining residents for meal and crowded dining rooms)  **Duration of int/ follow up**: 1year  **Compliance: N/A**  **Individualised: No**  **Ethics obtained: Yes** | **1º outcomes**:  Nutritional status (weight, BMI, Upper Arm Circumference)  Hydration status (appearing dehydrated)  Enjoyment of food/ drink  **2º outcomes**:  Cognitive status (MMSE)  Functional status (Grip strength)  GP or district nurse contact  **3º outcomes**  Mortality, chest infections, UTIs, falls | **BMI, mean (SD)**  Int:  1^st^ year 25.8(5.3),  2^nd^ year 25.6 (4.8)  Cont:  1^st^ year 24.7 (4.5),  2^nd^ year 24.3 (4.8)  **Weight (kg, mean, SD)**  Int:  1^st^ year 64.9(14.7),  2^nd^ year 64.9 (15.4)  Cont:  1^st^ year 62.3 (12.8),  2^nd^ year 61.5 (14.6)  **Enjoyment of food & drink (mean change, SD) using anonymous questionnaires**  Int +0.28 (0.43), Cont +0.09 (0.63)  P=0.24  **Residents appearing dehydrated (%, SD)**  Int:  1^st^ year 7 (23.2),  2^nd^ year 3 (10)  Cont:  1^st^ year 10 (38.5),  2^nd^ year 9 (34.6)  **No statistically significant effects** | **MMSE (mean, SD)**  Int:  1^st^ year 19 (5.6),  2^nd^ year 17 (6.2)  Cont:  1^st^ year 17 (6.2),  2^nd^ year 15 (7.9)  **No statistically significant effects** | **Mortality: n (%)**  Int: 11 (19%)  Cont: 10 (20%) |
| **Koss 1998**  **USA**  **Setting**  **High functioning dementia unit**  **Aim:**  To test the hypothesis that agitation may be triggered by a decrease in the amount of ambient light available rather than by an internal clock. | **Participants**: Self feeding dementia unit residents  **N:** 13  **M/F:** not reported  **Mean age**: NR  **Nutritional status: NR**  **Cognitive status: NR** **Dementia diagnosis:** diagnosed (no details)  **Dementia type:** AD  **Dementia stage:** NR  **Acute illness:** NR | **Design: BA**  **Int Type:** Environment (increased light intensity & enhancing visual stimulation during meals)  **Provided by: researcher?**  Details: 3 consecutive periods of 21 d each; baseline, int, post int. Same menu cycle in the 3 periods. Increased light intensity & enhancing visual stimulation during evening meals. Before each evening meal, tables positioned directly under existing lighting. Table set aiming at maximum contrast with white cloth, black & white place mats, coloured glasses and high contrast napkins. Usual setting was used for breakfast and lunch  **Cont**: usual setting  **Duration of int/follow up**: 21d int/ 63d study period  **Compliance:** measured but not reported  **Individualised: No**  **Ethics obtained: NR** | **1º outcomes**: none  **2º outcomes**:  Quantity of food intake (amount of food eaten)  **3º outcomes** |  | **Amount of food intake at dinner (recorded by staff after meal on a scale of 0-10, 0= nothing eaten & 10= everything eaten)**  Baseline (period 1): 6.57 (2.2)  Int (Period 2): 6.86 (2.3)  Post int (period 3): 6.36 (2.2)  **No statistical significance for P2 vs P1** (though P<0.03, higher intake at P2 compared to P3) |  |
| **McDaniel 2001**  **USA**  **Setting**  **Dementia unit**  **Aim:**  To evaluate noise and lighting conditions at mealtime and assess the food intake of ambulatory dementia residents living in Alzheimer’s unit. | **Participants**: Ambulatory residents of Alzheimer unit.  **N:** 16  **M/F:** 15/1  **Age range: 61-81**  **Nutritional status: NR**  **Cognitive status: MMSE range 0-24** **Dementia diagnosis: diagnosed (Alz unit)**  **Dementia type: AD**  **Dementia stage: various**  **Acute illness: NR** | **Design: BA**  **Int Type:** Dining environment  **Provided by: Researchers**  Details: Two 5-d phases conducted during wk one of the cycle menu. In 1^st^ phase participants ate breakfast and lunch in the Extended Care (EC) dining room (bigger, less noise, more lighting, TV and cafeteria style service). In the 2^nd^ phase, participants ate breakfast and lunch in the Alzheimer Unit (AU) dining room (smaller, relaxing music, more noise, less lighting, low ceiling and no cafeteria style)  **Cont**: N/A  **Duration of int/follow up**: 2wks  **Compliance: NR**  **Individualised: No**  **Ethics obtained: No** | **1º outcomes**:  Weight (measured but not reported)  **2º outcomes**:  energy, protein and  fluid intake  **3º outcomes** | **Weight:** no significant difference stated (3 participants lost >1 lb during EC phase compared to 2 in AU phase) | **Total breakfast and lunch calories over 5 d:** Kcal, mean  EC (phase 1): 5837 Kcal  AU (phase 2): 6167 Kcal  **Total breakfast and lunch fluid intake over 5 d:** ounces, mean  EC (phase 1): 113 ounce  AU (phase 2): 123 ounces  **Total breakfast and lunch protein over 5 d: grams, mean**  EC (phase 1): 230 g  AU (phase 2): 239 g  **No overall significant differences** (though some individual d or meals were sig diff.) |  |
| **Perivolaris 2006 (Period 1)**  **Canada**  **Setting**  **Cognitive support unit/ long term care facility**  **Aim:**  To evaluate an enhanced dining program to determine; its effects on residents’ intake, self-feeding abilities, and level of agitation, the extent that staff was able to implement the program and the degree of satisfaction of resident and staff participants of the program. | **Participants**: Self-feeding, dementia patients  **N:** 11  **M/F:** 8/3  **Mean age (range)**: 84.6 (77-93)  **Nutritional status: NR**  **Cognitive status : MMSE, mean (range) 13.9 (0-19)**  **Dementia diagnosis: diagnosed (no details)**  **Dementia type: 36% AD, 9% vascular, 54% unspecified**  **Dementia stage: Moderate to severe**  **Acute illness: NR** | **Design: BA** (repeated measures)  **Int Type:** 1. **Dining environment**, 2. Staff education  **Provided by: 1. Care home staff, 2. Researchers?**  Details: **Int 1:** wk 1-6: enhanced dining program:   - Home-like, bright, welcoming dining rooms (20-30 people). - Music played, aroma of bread and coffee, menu board. - Courses presented one at a time, on thermostore tray. - Staff using cues and prompts.   **Int 2**: “E” dining education program: introduced 6 wks after int.1. One d dining education workshop for staff to assist in providing meaningful dining experience for dementia patients.  **Cont**: N/A  **Duration of int/follow up**: 12wks  **Compliance:** NR  **Individualised:** No  **Ethics obtained: NR** | **1º outcomes**:  Measures of quality of life (level of agitation)  **2º outcomes**:  Quantity of food intake (energy intake)  Quality of food intake (feeding ability)  View of participants (resident satisfaction)  **3º outcomes** | **Level of agitation (Pittsburgh Agitation Scale):**  72% didn’t have these behaviours at any point, 1 maintained a low level of motor agitation and 1 improved after being mildly aggressive.  **Staff notes from focus group:** residents eating at a more leisurely pace, less wandering and more relaxed. | **Energy intake: Kcal**  Baseline: 490 Kcal  At 6 wks (int.1): 663 (P=0.005)  At 12 wks 677 (P=0.6)  **Feeding Ability Assessment: didn’t detect major variations.**  8/11 remained constant (7 were independent & 1 required 1 item at a time)  1/11 dramatically improved  1/11 independent at baseline & wk 6 but not at wk 12.  **Resident satisfaction: (n=8) (on a scale of 1-4)**  At 6 wks 3.25  At 12 wks 3.38 |  |
| **Ragneskog 1996**  **Sweden**  **Setting**  **Nursing home Psychogeriatric ward**  **Aim:**  To investigate whether dinner music influences food intake and symptoms common in dementia such as depressed mood, irritability and restlessness, as well as to ascertain whether a particular type of music was preferable. | **Participants**: Psychogeriatric ward residents  **N:** 24 allocated but 20 completed (4 died)  **M/F:** 10/10  **Mean age (range)**: 80 (69-94)  **Nutritional status: NR**  **Cognitive status: MMSE score,** 16 scored 0-10, 4 scored 11-24. **Dementia diagnosis: DSM-III & NINCDS-ARDRA criteria**  **Dementia type: mixed population**  **Dementia stage: 4 moderate, 16 severe.**  **Acute illness: NR** | **Design: BA** (repeated measures)  **Int Type:** Dining environment (dinner music)  **Provided by: NR**  Details: Five periods of study were included, with 3 phases of int which involved playing music of different types at mealtimes (from 5 mins before dinner until last patient had finished eating)  Phase 1 (no music 5 d)  Phase 2 Soothing music (8 d)  No activity (1 wk)  Phase 3 Tunes from 1920’s/30’s (10 d)  No activity 1 wk  Phase4 Pop music (8 d)  Phase 5 Cont (9 d)  **Cont**: N/A  **Duration of int/ follow up**: 26 int d/ 54 total study d  **Compliance:**  **Individualised: No**  **Ethics obtained: Yes** | **1º outcomes**: weight was measured but not reported  **2º outcomes**:  Quantity of food intake (amount consumed)  Overall reaction to music (sub-study: video observations of 26 meal situations of 5/20 patients)  Psychological wellbeing (GBS scale)  **3º outcomes**  Mortality | **? weight** | **Amount of consumed food, grams, mean (SD)**  No music  Total= 402 (100)  Soothing music  Total= 437 (102)  Music 20’s and 30’s  Total= 435 (98)  Pop music  Total= 459 (68)*  Cont  Total= 416 (74)  * p˂ 0.001  **GBS**  Significant differences in irritability , fear-panic and depressed mood (but not confusion, anxiety or restlessness) in all music periods combined compared to cont period (p˂0.05)  No effects on motor, intellectual or emotional impairment.  **Video observations:** (n=5) 4/5 patients spent more time (+22%, significance NR) eating dinner during the 3 int periods compared to cont. | **Mortality**  4/24 deaths |
| **Shatenstein 2000**  **Canada**  **Dementia unit of a nursing home**  **Aim:** To evaluate the nutritional and clinical consequences of changing from a centralised food delivery system to decentralised bulk food portioning. | **Participants**: Dementia Unit residents  **N:** 22  **M/F:** 1/21  **Mean age (SD)**: 81.6 (9.7)  **Nutritional status, BMI, mean (SD) 24 (4.0)**  **Cognitive status: NR**   **Dementia diagnosis: DSM IV criteria**  **Dementia type: AD, 68%; other types, 23%**  **Dementia stage: N/R**  **Acute illness: NR** | **Design: BA**  **Int Type:** Food service  **Provided by: care staff**  Details: Decentralised food service where meal portioning occurred on resident’s floor.  **Cont**: N/A  **Duration of int/ follow up**: 10wks  **Compliance: NR**  **Individualised: No**  **Ethics obtained: Yes** | **1º outcomes**:  Weight  BMI  Albumin  TST  MUAC  **2º outcomes**:  Proportion of food consumed  Energy Intake  **3º outcomes**  **none** | **Weight, kg: pre int 53.7 (10.4), post int 54.3 (SD: 10)**  **BMI,** kg/m^2^**: pre int 24 (4), post int 24 (3)**  **Albumin, g/l: pre int 35 (4), post int 33 (3)**  **TST, mm: pre int 14 (6), post int 16 (8)**  **MUAC, cms: pre int 25.7 (3.5), post int 25.9 (3.7)**  (none reported as significantly different from baseline, except that albumin was significantly lower at follow-up than baseline) | Proportion of food consumed, %: pre int 73 (14), post int 83 (9), p<0.04  Energy Intake, kcal: pre int 1555 (322), post int 1924 (373) P=0.0004  CHO and protein intakes also rose significantly |  |
| **Thomas 2009**  **US**  **Nursing home**  **Aim:**  **To examine whether music played during meals, by reducing agitation, would result in increased caloric consumption in nursing home residents with middle dementia.** | **Participants**: Residents of an AD unit within a dementia-specific, aged-care facility. Participants should have mid-stage AD, have adequate auditory skills, able to self-feed and be at risk of malnutrition.  **N:** 12  **M/F:** 1/11  **Mean age (range)**: 83.5 (76-92)  **Nutritional status: NR**  **Cognitive status: moderate dementia**   **Dementia diagnosis:** methods NR, but all participants resident in an AD unit of a dementia facility  **Dementia type:** AD  **Dementia stage:** middle stage as rated by the GDS scale for Assessment of Primary Degenerative Dementia  **Acute illness:** NR | **Design: BA (**time series)  **Int Type:** environmental  **Provided by: care staff and researchers**  Details: prior to int, set seating plan instituted and researchers spent 4 wks in dining area to become familiar to the residents. Int: music played during lunchtimes, alternate wks, at approx 60decibels. Music selection determined by researchers in consultation with families, likelihood of familiarity to majority of participants, variety of styles and consideration of tempo. Other distractions were removed (eg TV).  **Cont**: intervening wks no music was played.  **Duration of int/ follow up**: 8 wks  **Compliance: NR**  **Individualised: No**  **Ethics obtained: yes** | **1º outcomes**:  Meaningful activity  **2º outcomes**:  Quantity of food intake (amount consumed estimated as a proportion of amount served, reported in quartiles,(0, 25%, 50%, 75%, 100%)  **3º outcomes** | Meaningful activity (anecdotal results):  During music d, residents responded to music (dancing, foot tapping, body motions, increased facial animation), remained in dining area for longer and were more socially engaged. | **Energy intake, kcal/d, mean:**  Music wks: 797  Non-music wks: wks: 667  *(variance N/R, but results described as significant)* |  |
| **Van Ort 1995 Contextual int**  **US**  **Secure nursing unit**  **Aim:** To test the efficacy of two nursing ints, one contextual and one behavioural, designed to promote functional feeding and maintain adequate nutritional status of a sample of ‘demented’ elders in long-term care settings. | **Participants**: ‘non-combative’ residents from a secure unit within a larger residential geriatric centre, who were able to sit in a chair for meals, were responsive to human interaction, not usually restrained for eating, and required eating assistance.  **N:** 7  **M/F:** 2/5  **Age range**: 65-93  **Nutritional status:** NR  **Cognitive status:**  Severe dementia, MMSE, range: 0-2  **Dementia diagnosis:** residents of dementia unit, method of diagnosis N/R  **Dementia type:** N/R  **Dementia stage:** severe  **Acute illness:** NR | **Design: BA**  **Int**: **Contextual intervention (**Environment).  **Provided by: care staff**  Details:  **For 2 wks:**   - Noise and distractions minimised at mealtimes - Medications prepared away from dining area - All meals commenced in dining area - None were fed in reclining or ‘geri-chairs’ - Participants were seated between residents who could feed themselves - Disruptive residents were moved to alternative location - Meal was placed in front of resident on a placemat - Finger foods were provided, with bread being placed in each residents hand at the beginning of every meal. - Feeding assistants were not called away during meals   **Cont**: N/A  **Duration of int/ follow up**: 10wks (2 wks each int).  **Compliance:** NR  **Individualised:**  Contextual int: No  **Ethics obtained:** NR | **1º outcomes**:  Change in weight (measured but only reported as no change with no data provided)  **2º outcomes**:  Amount of food consumed.  Self-feeding behaviours.  **3º outcomes** | **Weight**  No change  **More sustained eating**--related interpersonal contact between residents and staff as shown by:  i). increased number of feeding cycles (defined as a feeding episode and associated feeding interval; feeding episode: uninterrupted series of feeding activities beginning with the feeder moving their hand towards resident’s mouth, completed bite or drink by the resident and feeder’s arm returning to baseline; feeding interval: activities which occur between the time the feeder’s arm returns to baseline and the beginning of the next episode).  ii). Decreased variability in length of the feeding intervals.  iii). Increased number of sustaining behaviours exhibited by feeders.  vi) Increased number of synchronised behaviour displayed by residents and feeders.  v). Decreased number interrupting/terminating behaviours displayed by feeders.  vi). Increased number of eliciting behaviours displayed by residents.  **I**mproved match between the functional abilities of the resident and the level of assistance offered by the feeder, as shown by:  i) Increased number of self-feeding behaviours by residents.  ii). Increased number of successful cues by feeders.  iii). Increased number of complex cycles (both residents and feeders engaged in food-related activities).  iv). Decreased number of aborted cues by feeders.  v). Decreased number of failed feeding attempts by the feeder.  Residents received more food and drink, less food was refused, more self-feeding occurred in the same amount of time.  Residents with less dementia interacted more with feeders. |  |  |
| **Wong 2008**  **(period 2 & 4)**  **New Zealand**  **Setting**  **Short stay assessment unit**  **Aim:**  To evaluate strategies designed to improve nutrition in elderly hospitalised patients with dementia. | **Participants**: Elderly dementia In-patients in a short stay unit  **N:** 98 (different patients in each of the 4 phases) P1:  n = 23, P2: n = 40, P3: n = 7, P4: n = 28.  **M/F:** 50/48 (P1: 12/11, P2: 17/23, P4: 16/12)  **Mean age (SD)**: P1: 80.0 (7.9), P2: 81.6 (9.7), P4: 80.4 (6.3)  **Nutritional status: BMI, mean (SD)** P1: 23.9 (3.7), P2: 23.2 (4.3), P4: 24.3(3.4)  **Cognitive status: NR** **Dementia diagnosis: dementia unit patients (no details)**  **Dementia type: NR**  **Dementia stage: NR**  **Acute illness: yes** | **Design: BA (**interrupted time series)  **Int Type:** P2 (food service), P4 (dining environment)  **Provided by: NR**  Details: Each period lasted 12 wks followed by a 5wk gap to ensure new patients in each.  **P2:** Encouraging dietary ‘grazing’ by; providing a glass door fridge filled with snacks and beverages accessible to patients 24 hrs/d and meals at an earlier time for those requiring more assistance or more time.  **P4:** Improving the ambience of the dining area by playing soothing music at meal times.  **Cont**: P1 observation only  **Duration of int/ follow up**: 12 wks each int (total 36 wks)  **Compliance: NR**  **Individualised: No**  **Ethics obtained: Yes** | **1º outcomes**:  Nutritional status (BMI, Mid arm circumference (MAC))  **2º outcomes**:  Quantity of food intake (energy intake) not presented for all phases/ not compared to baseline  **3º outcomes** | **BMI, Kg/m2, mean change:**  P1: -0.6 (P<0.001).  P2: +0.3 (p<0.04),  P4: +0.39 (p<0.007)  **Mid arm circumference (MAC), cm, mean (SD)**  P1: not measured  P2: + 0.04 (0.07),  P4: 0.09 (0.44)  (all NS) | **Energy intake, Kcal, mean change**  P4: +129.2 (91.4) (compared to P2) |  |

**Supplemental Table 2. Characteristics and results of education and training interventions (EDWINA)**

| **Study** | **Participants** | **Interventions** | **Outcomes*** | **1° outcomes*** | **2° outcomes*** | **3° outcomes*** |
| --- | --- | --- | --- | --- | --- | --- |
| **Altus 2002**  **(period 2)**  **USA**  **Setting:**  **Locked dementia unit**  **Aim:**  To examine if changing the mode of meal delivery to “family-style” would increase residents’ communication and participation in mealtime tasks. | **Participants**: Dementia unit residents  **N:** 5  **M/F:** 0/5  **Mean age (range)**: 80 (76-87)  **Nutritional status: NR**  **Cognitive status: mean** MMSE score (range) 8 (3-16)  **Dementia diagnosis:** physician diagnosis  **Dementia type:** AD or other dementia  **Dementia stage:** Moderate to severe  **Acute illness:** NR | **Design:** BA (time-series repeated measures)  **Int**: Family- style meals (with or without nursing assistant training)  **Details: ABAB’ design**  **Provided by:** health worker/care home staff  Details: **A** (baseline 1, 10 d) **B** (Int 1, family style meals 5 d) **A** (baseline 2, 5d) **B’** (Int 2, family style + assistant training 5d)  **Cont**: pre-plated meals  **Duration of int/ follow up**: 5d i  **Compliance:** NR  **Individualised: No**  **Ethics obtained: NR** | **1º outcomes**:  Meaningful activity (% of resident participation in mealtime, appropriate & inappropriate communication, frequency of praise by nurse)  **2° outcomes**:  Views or attitudes of participants  **3° outcomes** | **Resident participation in mealtime tasks: (% of participation)**  24% after int 1 vs 10% baseline 1  65% after int 2 vs 6% baseline 2  **Appropriate communication:** (mean % of intervals with appropriate communication)  10.6% after int 1 vs 5.5% baseline 1  17.9% after int 2 vs 3.8% baseline 2  **Praise statements:** (frequency of praise statements by CNA**)**  7.2/meal post int 1 vs 0.2/meal baseline 1  14.2/meal post int 2 vs 0/meal baseline 2  **Suggested improvements** in int 2 vs int 1 but no formal significance testing | **CNA’s satisfaction with level of participation:**  post int: 5 (very satisfied) vs pre int: 2 (somewhat satisfied) |  |
| **Aselage 2011**  **USA**  **Setting:**  **Nursing home**  **Aim:**  To test the clinical feasibility of an int to train NH staff via a web-based dementia feeding skills. The goal was to alleviate mealtime difficulties in persons with dementia who require assistance | **Participants**: NH residents diagnosed with dementia  **N:** 10 (7 analysed, 4 int & 3 cont))  **M/F:** not reported  **Mean age (SD)**: NR  **Nutritional status:** NR  **Cognitive status:** MMSE score <19 **Dementia diagnosis:** diagnosed (no details)  **Dementia type:** NR  **Dementia stage:** Moderate  **Acute illness:** NR | **Design:** RCT parallel (cluster)  **Int Type:** Staff education  **Provided by:** Researchers  Details: 17 staff viewed a feeding skills web-based training module with a pre and post-test of knowledge. 3x1hr coaching sessions delivered at Wk3 & Wk5. Pre int tests completed by both groups.  **Cont**: Usual care  **Duration of int/ follow up**: 2mo  **Compliance: NR**  **Individualised: No**  **Ethics obtained: Yes** | **1º outcomes**:  Weight (from records), Quality of life (assessed by NH staff, score 13-52 with higher scores indicating better QoL)  **2º outcomes**:  Quantity of food intake (% of food consumed, meal obs made on 3 weekends), time spent feeding, EdFed (Edinburgh Feeding Evaluation in Dementia scale: scores 0-20, higher scores indicate greater impairment)  **3º outcomes: none** | **Body weight, lb: (SDs NR)**  Baseline: int 121.9 vs cont 131.3  1 mo: int. 122.5 vs cont 132.3  **Quality of life: mean (range)**  Baseline: int.20.25 (14-28) vs cont 20.33 (18-22)  8wks: int.11.25 (7-15) vs cont 19 (14-22) | **Food intake: (% food consumed)**  Baseline: int.6.8 vs cont 29.7  8 wks: int 18.4 vs cont 13.2  **Time spent eating:**  Baseline 27 min 41 sec int. vs 24 min 8 sec cont  8 wks: 35 min 15 sec int. vs 14 min 38 sec cont  **Ed-FED**  Baseline: int.7 vs cont 4.8  8 wks: int.8.7 vs cont 6.5 |  |
| **Faxen-Irving**  **2002**  **Sweden**  **Setting**  **Group-living for demented people**  **Aim:**  To study the effects of nutritional int on body weight, cognition and ADL function in demented individuals. | **Participants**: people diagnosed with dementia living in two units  **N:** 36 (22 int unit, 14 cont unit) 33 analysed  **M/F:** 2/31  **Mean age (SD)**: int. 83 (4), cont 85 (4)  **Nutritional status**  **Cognitive status: MMSE, mean (SD)** int.9 (6.6), cont 8.5 (6.2)  **Dementia diagnosis: diagnosed (no details)**  **Dementia type: AD (22%), vascular (20%), unspecified (58%)**  **Dementia stage: 12 mild, 10 moderate, 12 severe & 1 questionable.**  **Acute illness: NR** | **Design: cluster CCT**  **Int Type:** Nutritional & educational int  **Provided by: care staff**  Details:  * 2 x 200 ml ONS daily for 5 mo; a juicy supplement (170 kcal) in between meals in the afternoon and a balanced supplement (240 kcal) in the evening.  * Education: staff attended 12 hr program about nutrition & diet for the elderly given by dieticians, physicians and external care personnel. Lectures were combined with practical exercises e.g. calculating BMI, thickening or enriching drinks.  **Cont**: usual care (no further details)  **Compliance:** 76% participants consumed all, rest consumed about half.  **Individualised: No**  **Duration of int/ follow up**: 5 mo  **Ethics obtained: Yes** | **1º outcomes**:  Nutritional status (BMI, weight, TSF, AMC, Nutrition score NuSc, serum albumin, serum haemoglobin)  **2º outcomes**:  Measures of functional status (ADL)  Measures of cognitive status (CDR Clinical Dementia Rating Scale, MMSE)  **3º outcomes** | **Body weight (kg, mean & SD)**  Int: Baseline 55.4 (10.4), 6 mo 58.8 (11.2)  Cont: baseline 62.2 (8.2), 6 mo 61.9 (10.4)  **P=0.003** (difference in change between groups)  (9mo after supplementation stopped, weight decreased by 2.6(3) kg in the remaining 15 residents of the int unit) p<0.1  **BMI (mean, SD)**  Int: Baseline 22.2 (4.1), 6 mo 23.5 (4.4)  Cont: baseline 24.6 (2.9), 6 mo 24.5 (3.8)  **P=0.003 (difference in change between groups)**  **Serum albumin and haemoglobin, arm muscle circumference, and risk of malnutrition (by NuSc)** worsened in both int and cont groups (no significant different suggested)  **TSF** improved in int and worsened in cont, p=0.002 between groups | **ADL (activities of daily living A-G, median, range)**  Int: baseline E (A-G), 6 mo F (B-G)  Cont: baseline D (A-G), 6 mo E (B-G)  p>0.05  **MMSE**  Int: Baseline 10.9 (6.2), 6 mo 8.4 (5.9)  Cont: baseline 10.2 (5.3), 6 mo 10.7 (5.3)  P=0.01  **Clinical Dementia Rating, CDR total score (0-18)**  Int: Baseline 12.3 (5), 6 mo 14.7 (3.6)  Cont: baseline 11.4 (4.6), 6 mo 13.3 (3.6)  p>0.05 |  |
| **Jean 1997**  **USA**  **Setting: Nursing home**  **Aim:** To implement a finger food menu with selected residents to determine whether this could improve their overall nutritional status and independent feeding skills. | **Participants:** cognitively impaired NH residents with poor intake and limited use of eating utensils  **N: 12**  **M/F: NR**  **Mean age(SD): NR**  **Nutritional status:** had weight loss (no details)  **Cognitive status: c**ognitively impaired (no data) **Dementia diagnosis: NR**  **Dementia type:** half had AD & half other dementias**.**  **Dementia stage: NR**  **Acute illness: NR** | **Design: BA**  **Int Type: Finger food menu plus staff education**  **Provided by: health worker**  **Details:** four-wk cycle menu. Training for nursing and dietary staff on the rationale for the finger food diet including fundamental concepts of the menu alterations and actual presentation of the meals  **Cont: N/A**  **Duration of int/ follow up: 6mo**  **Compliance: NR**  **Individualised: No**  **Ethics obtained: no** | **Weight loss arrest (number of participants with weight loss arrest after 6 mo)**  10/12 participants  **Discontinuation of supplements:**  In 25% of cases it was found that high calorie and protein supplements could be discontinued.  **Numeric data lacking, statistical significance unclear for all outcomes** | **Feeding skills independence:** (a scale to measure feeding assistance)  All of the residents became more independent with feeding skills. 3 patients who initially required feeding assistance were able to feed themselves entirely.  **Numeric data lacking, statistical significance unclear for all outcomes** |  |  |
| **Mamhidir 2007**  **Sweden**  **Setting: Nursing homes**  **Aim:**  To follow weight changes in patients with moderate and severe dementia and analyse how these changes related to biological and psychological parameters after staff education and support in integrity promoting care. Also to describe meal environment and routine. | **Participants: residents with dementia from two different nursing home wards**  **N: 33 (18 int, 15 cont)**  **M/F: 10/23**  **Mean age (SD): 82 (6.3)**  **Nutritional status: NR**  **Cognitive status Dementia diagnosis: NINCDS-ADRDA for AD, DSM-III for multi infarct dementia**  **Dementia type: 19 AD, 12 multi-infarct, 2 others**  **Dementia stage: 17 severe, 3 moderate, 5 mild, 1 questionable.**  **Acute illness: NR** | **Design: CCT**  **Int Type: Staff training**  **Provided by: Researchers and health workers**  **Details:** One wk course for all staff by a psychologist and a nursing science professor. Lectures 20 hrs and group discussions 18 hrs focused on human relationships, normal aging, environment, communication, interaction, integrity, confusion and dementia disorders. Followed by 3 mo extensive support- RA based on ward, nurse researcher visited ward 3-4 d/ wk. Lectures video recorded for repeat viewing. Care was video recorded and used in discussions of care delivery. Staff kept diaries of changes to routines and environment etc. Patients served on trays at beginning- moved to serving from dishes at tables.  **Cont: usual care.** Both wards had meals provided from same centralised kitchens- so had same food.  **Duration of int/ follow up: 3mo**  **Compliance: NR**  **Individualised: No**  **Ethics obtained: Yes** | **1º outcomes: Nutritional status (weight)**  **Meaningful activity (mealtime environment and routines)**  **2º outcomes:**  **3º outcomes** | **Change in weight at 4 mo: Kg, mean (SD)**  Int: 0.53 (3.7)  Cont: -4.1 (5.03)  P<0.01  Weight increased in 13/18 int and decreased in 13/15 cont  **Mealtime environment and routines (analysis of staff diaries)**  Int: Food served in bowls- so patient could help themselves, increased contact between patients and staff, more pleasant atmosphere.  Environment more homelike- pictures on dining room walls, tablecloths and curtains chosen by staff with patients, private possessions brought into home, staff wore brightly coloured clothes.  Cont: No changes were made to routines/ environment |  |  |
| **Mentes 2003**  **USA**  **Setting: Nursing homes**  **Aim:**  To evaluate the hydration management guideline with respect to its efficacy in long term care settings. | **Participants: Cognitively impaired nursing home residents**  **N: 8**  **M/F: all females**  **Mean age: 89**  **Nutritional status: NR**  **Cognitive status:** MMSE, mean (SD) 18 (4.7) (range 11-24) **Dementia diagnosis:** NR  **Dementia type:** NR  **Dementia stage:** mostly mild?  **Acute illness:** NR | **Design: BA**  **Int Type: Hydration management**  **Provided by: Research nurse**  **Details:**  **Wk 1:** risk factor identification (comprehensive assessment) and individual daily plan to prompt adequate hydration.  Wk 2-4: oral hydration management with calculation of fluid goal adjusted for weight 100mL/ Kg for 1st 10 Kgs, 50 ml/ Kg for 2nd 10 Kgs and 15 ml/ Kg for remaining.  **Cont:** NR  **Duration of int/follow up:** 4wk  **Compliance:** NR  **Individualised:** Yes  **Ethics obtained: Yes** | **1º outcomes:**  **Hydration status (specific gravity)**  **2º outcomes:**  **Quantity of fluid intake**  **View of staff**  **3º outcomes** | **Urine specific gravity: mean (SD)**  Baseline: 1.017 (0.008), end 1.018 (0.006). Change +0.001 (0.008)  p>0.05 | **24 hour Fluid intake, ml, mean (SD)**  Baseline: 1437 (542),  End: 1503 (426).  Change +66 (210)  **Staff views:**  During interviews with staff to identify areas for improving hydration management guidelines staff commented on patient access and independence, patient cooperation and staff motivation. |  |
| **Suominen 2007**  **Finland**  **Nursing home**  **Aim:**  **To develop nutrition education for professionals in dementia wards to evaluate the effects of education and determine the outcome of the education on the nutrition of aged residents.** | **Participants: Staff and residents of specialist dementia units in 5 nursing homes**  **N: 28 staff (23 nurses, 5 food personnel), 21 residents**  **M/F: Residents: 0/21**  **Mean age (range): residents: 85 (62-95)**  **Nutritional status, mean energy intake, kcal/resident/d: 1230 (variance NR)**  **Cognitive status Moderate to severe Dementia diagnosis: residents on dementia units, but method of diagnosis N/R**  **Dementia type: NR**  **Dementia stage: moderate - severe**  **Acute illness: NR** | **Design: BA**  **Int Type: educational**  **Provided by: research nutritionist**  **Details**: 6 training sessions over 6 mo lasting (2-3hrs). Following session 1 staff assessed their residents with MNA; after session 2 3d weighed intake and food diaries completed, analysed data using computer-based food analysis programme and with guidance from the nutritionists, they discussed planned dietary changes for individual residents.  **Cont: N/A**  **Duration of int/ follow up: 12 mo**  **Compliance: Not measured**  **Individualised: yes**  **Ethics obtained: NR** | **1º outcomes:**  **Weight**  **BMI**  **MNA**  **2º outcomes:**  **Energy intake**  **Protein**  **Views of participants**  **3º outcomes**  **none** | **Weight:** 42% increased weight; 42% lost weight  **BMI, mean, kg/m2:**  Before: 21.7  After: 21.4;  (variance and p values NR)  **MNA, at risk of malnutrition,**  Before: n=17/19  After: n=12/19  **Malnourished,**  Before: n=2/19  After: n=4/19  p=0.10 | **Energy intake, mean, kcal/resident/d:**  Before: 1230  After: 1487; p≤0.001  **Protein, mean, g/d:**  Before: 50.4  After: 60.9; p=0.006  (no measures of variance reported)  **Staff Views:**  Staff found filling in food diaries and discussing results with nutritionist most helpful in learning about nutritional care of residents. MNA also useful. Staff surprised at how little energy patients consumed prior to int and did not understand link between nutritional status and well-being. Weighing food portions laborious, though helpful. Staff became motivated, confident in assessing nutritional needs and instituting changes. |  |
| **Wikby 2009**  **Sweden**  **Setting: Residential care**  **Aim:** To test the hypothesis that education provided to staff regarding nutritional needs and individualizing nutritional care will improve the nutritional status and functional capacity of elderly people newly admitted to resident homes. | **Participants: Newly admitted residents from 8 nursing homes**  **N: 127 allocated, 115 analysed (8 NHs) 3 NHs (n=68) int., 5 NHs (n=59) cont**  **M/F: 34/81**  **Mean age (SD):** 85.5 (6.1) int., 85.2 (6.5) cont.  **Nutritional status: NR**  **Cognitive status:** MMSE, mean (range) int. 17 (6-22), cont 12 (0-21).  **Dementia diagnosis: NR**  **Dementia type: NR**  **Dementia stage: NR**  **Acute illness: NR** | **Design: CCT**  **Int Type:** Staff training  **Provided by:** Research team  **Details:** 4 steps  1. Info to staff about study and a book on food and dietary management in elderly people. 2. Info on managing a study circle and identifying individual needs. 3. Nutritional problems discussed in study circles (5 afternoons for 3 mo). 4. Staff assessed nutritional status in newly admitted residents using the MNA and told to predict the energy requirement, and design a nutritional programme.  **Cont: No education**  **Duration of int/ follow up: 4mo**  **Compliance: NR**  **Individualised: No (staff trained to individualise care)**  **Ethics obtained: Yes** | **1º outcomes:**  **Nutritional status, including weight, weight index, Triceps Skinfold Thickness (TSF), Protein energy malnutrition (PEM), Arm muscle circumference (AMC))**  **2º outcomes:**  **Cognitive status (MMSE)**  **Functional status (ADL, Activity index)**  **3º outcomes** | **Weight, Kg, mean (SD)**  Int Baseline: 61.4 (10.3), 4 mo. 62.8 (9.7)  Cont: Baseline: 59.5 (11.4), 4 mo. 60.7 (11.7)  **Weight index,** % of reference weight, mean (SD)  Int Baseline: 94.8 (15.5), 4 mo. 97.2 (15.2)  Cont: Baseline: 92.0 (14.8), 4 mo. 93.9 (14.7)  P=0.24  **TSF, mm, mean (SD)**  Int Baseline: 12.4 (5.0), 4 mo. 13.1 (5.3)  Cont: Baseline: 11.2 (5.4), 4 mo. 12.1 (5.6)  P=0.31  **PEM, number**  Int Baseline: 20, 4 mo. 7  Cont: Baseline: 17, 4 mo. 10 P=0.51  **AMC, cm, mean (SD)**  Int Baseline: 23.2 (2.9), 4 mo. 23.6 (2.8)  Cont: Baseline: 22.6 (2.5), 4 mo. 22.9(2.3)  P=0.16 | **ADL, score: median (IQ range):**  Int Baseline: 27 (22-34), 4 mo. 29 (22-36)  Cont: Baseline: 23 (16-28), 4 mo. 24 (16-32) P=0.035  **MMSE, score: median (IQ range):**  Int Baseline: 17 (6-22), 4 mo. 19 (6-24)  Cont: Baseline: 12 (0-21), 4 mo. 11 (0-21)  P=0.03 |  |
| **Riviere 2001**  **France, Italy & Spain**  **Setting**  **Living at home with informal carer**  **Aim:**  **To determine if a nutritional education program can prevent weight loss in AD patients.** | **Participants: AD patients living at home from 3 European cities**  **N: 225 (151 int., 74 cont)**  **M/F: 74/151**  **Mean age (SD):77.3 (8.2) int., 75.4 (7.9) cont**  **Nutritional status: MNA, mean (SD) int. 22.9 (3.7), cont 24.3 (2.9)**  **Cognitive status  Dementia diagnosis: NINCDS-ADRDA criteria**  **Dementia type: AD**  **Dementia stage: NR**  **Acute illness: NR** | **Design: CCT**  **Int Type: Education (to caregivers)**  **Provided by: Health workers**  **Details:** Caregivers were offered 9 x 1 hour nutritional sessions over a year in groups of ~10. Five were in the 1st mo and 1 each in mo 2, 3, 6 & 12. Each session dealt with particular topic like enriching food, combating eating behaviour disorder etc. presented by dietician or another health professional. They also received a calendar containing nutritional advice and to record monthly weight  **Cont:** No sessions but caregivers were offered advice provided in normal follow up.  **Duration of int/ follow up: 1 year**  **Compliance: participants had to attend a minimum of 5/10 sessions**  **Individualised: No**  **Ethics obtained: NR** | **1º outcomes:**  **Nutritional status (weight, MNA)**  **2º outcomes:**  **Functional status (ADL)**  **Cognitive status (MMSE)**  **3º outcomes** | **Weight change from 0-12 mo, Kg, mean (SD)**  Int: +0.7 (3.6)  Cont: -0.7 (5.4)  P<0.05  **MNA change at 12 mo, mean (SD)**  Int: +0.3 (2.6)  Cont: -1.0 (3.4)  P<0.005 | **ADL change from 0-12 mo, mean (SD)**  Int: -0.7 (1.4)  Cont: -0.7 (1.2)  **MMSE change from 0-12 mo, mean (SD)**  Int: -3.2 (3.1)  Cont: -0.7 (1.2)  (reviewers analysis: p<0.0001) |  |
| **Hanson 2010**  **USA**  **Setting**  **Nursing homes**  **Aim:**  To test whether a decision aid improves quality of decision making about feeding options in advanced dementia | **Participants**: Advanced dementia patients with feeding problems from 24 NHs  **N:** 256 surrogate-resident dyads randomised (127 int., 129 cont) NHs randomised in pairs matched on variables ass with tube feeding rates among others.  **M/F:** 58/198  **Mean age**: 85.2 int., 85.3 cont  **Nutritional status:** weight loss 9% int., 12% cont. Poor intake 44% int., 52% cont.  **Cognitive status: Cog Performance scale in MDS (0-19)** 4.44 int., 4.14 cont **Dementia diagnosis:** chart diagnosis confirmed with GDS by the shift nurse  **Dementia type: NR**  **Dementia stage: severe**  **Acute illness: NR** | **Design: cluster RCT parallel**  **Int Type:** Education (decision aide)  **Provided by:** research staff  **Details:** Surrogates in the int sites received a structured decision aid providing information about dementia, feeding options & the outcomes, advantages & disadvantages of feeding tubes, taking an average of 20 min. Surrogates received the print aid to take home and researchers prompted them to discuss it with healthcare providers.  **Cont**: Usual care including information from healthcare providers.  **Duration of int/ follow up**: tool 20 min but 3&9 mo follow up  **Compliance:** NR  **Individualised: No**  **Ethics obtained: Yes** | **1º outcomes**:  Nutritional status (weight loss)  **2º outcomes**:  Views or attitudes of carers  **3º outcomes**  Mortality | **% with weight loss:**  Baseline: 9% int., 12% cont  at 9 mo: 6% int., 16% cont p=0.01 | **Effect of decision aide on knowledge, expectation & treatment preferences:** (done for int arm only)  **Knowledge score (0-19)** 15.5 pre vs 16.8 post p<0.001  **Levels of decisional conflict following decision aid** (2.24  pre vs. 1.91 post, p<0.001)  **Rating of certainty about their choice of oral feeding** (1.35 pre vs. 1.05 post ; p = 0.016). | **Mortality:**  Baseline to 9 mo  34/127 27% int, 37/127 29% cont. |
| **Kwok 2012**  **Hong Kong**  **Setting**  **Old age hostels**  **Aim:**  To examine whether dietary ints promote intake of fruit, veg, fish and lower salt intake were effective in preventing cognitive decline in older people. | **Participants**: over 75yrs hostel residents.  **N:** 14 hostels (429 participants,) 204 int from 6 hostels (120 with MCI) & 225 (149 with MCI) cont from 8 hostels.  **M/F:** 66/363  **Mean age (SD)**: 83 (5.7) int, 83 (5.5) cont.  **Nutritional status: BMI, mean (SD) int.** 23.4 (4), cont 23.6 (4)  **Cognitive status: MMSE, mean (SD) int.** 23.1 (3.9), cont 22.4 (4.6). **CDR** 0.5 (0.5, 1.5) int, 1.0 (0.1, 1.5) cont. **Dementia diagnosis:** int. 120 (58.8%), cont 149 (66.2%) with Questionable dementia (QD) based on CDR  **Dementia type: MCI**  **Dementia stage: N/A**  **Acute illness: NR** | **Design: RCT parallel**  **Int Type:** Education  **Provided by: Dietitian**  Details: 1. One hour talk by PI & research dietician to residents & staff on dementia prevention and promotion of “brain preservation diet”.  2. Dietary support groups by a trained dietitian (10-15 in group). The groups met once x 3wks (45 min each) for the 1^st^ year reduced to 1 x 6wks for mo 13-33.  3. Each session had a specific theme related to health eating and preservation diet.  4. Dietitians liaised with staff on menu and cooking methods.  5. Individual dietary counselling for people with chewing problems and staff were advised to modify food.  **Cont**: The cont group had the first element of the int only (i.e. the 1 hour talk)  **Duration of int/ follow up**: 33 mo  **Compliance:** attendance rate 79.6% 1^st^ year, 71.5% subsequently  **Individualised: yes**  **Ethics obtained: NR** | **1º outcomes:**  Weight  **2º outcomes:**  Quantity of food intake  Cognitive status measures (CDR)  **3º outcomes:**  Mortality | **Weight:**  “no significant group differences in changes in body weight” but no results reported | **Changes in veg. intake (mean portion/d, SD)**  (all outcomes only extracted for people with MCI at baseline)  **Change at 12 mo.**  0.3 (1.2) int n=115, 0.1 (1.1) cont n=138  at 24 mo. -0.3 (1.4) int n=80, -0.2 (1.4) cont n=94  at33 mo. -0.2 (1.2) int n=66, -0.3 (1.2) cont n=76  **Changes in fruit intake (mean portion/d, SD)**  **Change at 12 mo.**  0.2 (1.1) int n=117, -0.2 (1.0) cont n=139*  24 mo. -0.2 (1.3) int n=82, -0.1 (1.3) cont n=96  33 mo. -0.3 (1.3) int n=68, -0.6 (1.3) cont n=77  **Changes in fish intake (mean portion/d, SD)**  **Change at 12 mo.**  **-0.3 (2.8) int n=110, -0.1 (2.6) cont n=132**  **at 24 mo. -1.1 (3.3) int n=77, -3.3 (4.9) cont n=93***  **at33 mo. -1.8 (2.8) int n=65, -3.2 (4) cont n=77**  ***p= <0.05**  **Cognitive decline** (n, %)  at 24 mo: 11 (12%) int n=92, 20 (16.7%) cont n=120.  at 33 mo: 18(19.8%) int **n=91,** 27 (23%) cont n=117. | **Mortality:**  **at 24 mo: 19/204 int, 17/225 cont**  **at 33 mo: 8 int, 8 cont** |
| **Pivi 2011**  **Education arm**  **Brazil**  **Setting**  **NR**  **Aim:**  To evaluate if there is a difference between nutrition education and oral nutritional supplementation on nutritional status in patients with AD. | **Participants**: Elderly patients with probable AD  **N:** 78 (Education 25, supplement 26, cont 27)  **M/F:** 25/53  **Mean age**: 75.2  **Nutritional status: NR**  **Cognitive status: MMSE, mean** 12.8 (Edu), 11.6 (suppl), 12.6 (cont **Dementia diagnosis: DSM-IV and CDR**  **Dementia type: AD**  **Dementia stage: 23 mild, 31 moderate, 24 severe (based on CDR)**  **Acute illness: NR** | **Design: RCT parallel (3 arms)**  **Int Type:** 1. Education,  **Provided by:**  Details: **Education group (EG):** patients and caregivers participated in educational program consisting of 10 classes. Each class had a maximum of 10 participants with the aim of greater interaction. Each class was supported by slides in accordance with the Brazilian Association of Alzheimer’s. The classes were developed with relevant topics to the needs of nutritional int.  **Cont**: usual care plus monthly nutritional assessment.  **Duration of int/ follow up**: 6 mo  **Compliance:** NR  **Individualised:** No  **Ethics obtained: Yes** | **1º outcomes**:  BMI, weight, Arm circumference, Triceps skinfold (TSF)  (statistics and variances are unclear as are time points)  **2º outcomes**  **3º outcomes** | **BMI, change after 6 mo: mean**  Int: +1.2, cont: -2.2  p>0.05?  **Weight change, mean Kg**  Int: 1.2, cont: -2.2  p>0.05?  **Arm circumference, cm**  Int: 1.9, Cont: -0.4  p>0.05?  **Triceps skinfold (TSF), mm**  Int: 2.3, Cont: 2.2  p>0.05  **Albumin, units unclear**  Int: -4.4, Cont: -3.2  p>0.05 |  |  |
| **NutriAlz Trial**  **Salva 2009**  **Spain**  **11 outpatient clinics and hospital day-care centres**  **Aim:** To assess effectiveness of health and nutrition programme (NutriAlz) versus usual care on functional level in older people living with dementia at home, as well as on clinical practice related to nutrition and the care-giver’s burden | **Participants:**  **N: 946 (656, 69% completed follow-up)**  **M/F: Int. 148/300; Cont 154/344**  **Mean age (SD): Int. 79.4 (7), Cont 78.6 (7.5)**  **Nutritional status, BMI, mean (SD) Int. 26.6 (4.4), cont 27.3 (4.6)**  **Cognitive status, MMSE, CDR, mean (SD):**  **Int:**  **MMSE: 14.7 (6.0), CDR: 1.8 (0.8)**  **Cont: MMSE: 16.0 (6.25), CDR: 1.7 (0.8)**  **Dementia diagnosis:** DSM IV criteria  **Dementia type:** AD, vascular dementia, mixed and other types  **Dementia stage:** mild, moderate  **Acute illness: NR** | **Design: RCT**  **Int Type: Educational**  **Provided by:** self, family carer, health worker  **Details:**  1. Standardised protocols for feeding and nutrition for HP’s.  2. Four training sessions for people with dementia and their family carers by dietitian.  3. Programme newsletter.  4. Support for weight monitoring.  **Cont:** usual care (not specified)  **Duration of int/follow up:** 12mo  **Compliance: NR**  **Individualised: No**  **Ethics obtained: Yes** | **1º outcomes:**  **Nutritional status, weight, BMI, MNA, EBS (Eating Behaviour Scale)**  **2º outcomes:**  **Functional status, ADL**  **Cognitive status, MMSE, CDR**  **Zarit Score (Caregiver burden)**  **3º outcomes**  **Mortality** | **Weight, mean change:** Int 0.26 (95%CI: -0.57, 1.09)  Cont 0.09 (95%CI: -0.7, 0.52); p=0.60  **BMI, mean change:**  Int -0.01 (-0.21, 0.19)  Cont -0.06 (-0.22, 0.22); p=0.84  **MNA, mean change:**  Int 0.46 (0.09, 0.83)  Cont 0.66 (-0.80, 0.21); p=0.03  **EBS, mean change:**  Int -1.65 (-2.04, -1.28)  Cont 1.24 (-1.61, -0.87); p=0.70  **Zarit score, mean change:**  Int 0.59 (-0.99, 2.17)  Cont 2.36 (1.26, 3.46); p=0.68 | **ADL, mean change:** Int: -0.83 (-0.69, -0.97)  Cont -0.74 (-0.62, -0.86); p=0.95  **MMSE, mean change:**  Int -2.21 (-2.68, -1.74)  Cont -2.21 (-2.60, -1.82); p=0.95  **CDR, mean change:** Int 0.35 (0.29, 0.41)  Cont: 0.35 (0.29, 0.41); p=0.69 | **Mortality: 72 (8%) across both groups, (no observed differences reported)** |
| **Suominen 2013**  **Finland**  **Setting: Community-dwelling**  **Aim**: to investigate whether tailored individualised nutritional counselling and care have an effect on weight, nutrition, functioning and QOL of AD patients and their spouses**.** | **Participants**: Aged (>64 years) AD patients and their spouses living at the same address in the Helsinki area and able to travel to the study centre.  **N:** 101 couples (50 int group)  **M/F:** 69% AD patients were male  **Mean age (SD)**: Int. 77.4 (5.6), Cont NR  **Nutritional status, protein, g/body weight/d, mean (variance N/R):**  Int. 0.97, Cont 1.00  **Cognitive status, MMSE, mean (SD):**  Int. 19.3 (5.6), Cont NR  **Dementia diagnosis:** retrieved from AD medication register, Finland  **Dementia type: AD**  **Dementia stage:** as determined by MMSE  **Acute illness:** NR | **Design:** parallel RCT  **Int Type:** individualised nutritional care, educational  **Provided by: nutritionist**  Details: Nutritionist instructed couples on how to keep a 3-d food diary. Analysed using Nutrica program. During 4-8 visits, the nutritionist aimed to correct any nutrient deficiencies by recommending dietary changes or use of supplements. Couples were prescribed oral vitamin D (20μg/d), received oral and written advice regarding exercise to strengthen muscles and could also attend a nutritional support group.  **Cont**: Following randomisation, couples were provided with a written guide on the nutrition of older people. On completion of the trial, they received 2 further handbooks on good nutrition and good sources of protein for aged people.  **Duration of int/ follow up**: 12 mo  **Compliance: NR**  **Individualised: yes**  **Ethics obtained: yes** | **1º outcomes**:  Weight  QOL  **2º outcomes**:  Protein intake  **3º outcomes** | **Weight:** “no difference in weight change between the groups after 1-year follow-up” reported (preliminary results from a conference abstract)  **QOL:** described as improved in the int group | **Protein, g/body weight/d, mean (95%CI)**  *Int:* 1.04 (0.97, 1.11)  *Cont:* 0.92 (0.85,0.99); p=0.03 |  |
| **Perivolaris 2006**  **E dining education**  **Canada**  **Setting: Cognitive support unit in LTC facility**  **Aim:** To evaluate effects of an enhanced dining program on residents’ intake, self-feeding abilities etc | **Participants**: Self-feeding, dementia patients  **N:** 11  **M/F:** 8/3  **Mean age (range)**: 84.6 (77-93)  **Nutritional status: NR**  **Cognitive status : MMSE, mean (range) 13.9 (0-19)**  **Dementia diagnosis: diagnosed (no details)**  **Dementia type: 36% AD, 9% vascular, 54% unspecified**  **Dementia stage: Moderate to severe**  **Acute illness: NR** | **Design: BA** (repeated measures)  **Int Type:** Staff education  **Provided by: Researchers?**  Details: “E” dining education program (Period 2): introduced 6 wks after int.1. One d dining education workshop for staff to assist in providing meaningful dining experience for dementia patients.  **Cont**: N/A  **Duration of int/ follow up**: 12 wks  **Compliance:** NR  **Individualised:** No  **Ethics obtained: NR** | **1º outcomes**:  Measures of quality of life (level of agitation)  **2º outcomes**:  Quantity of food intake (energy intake)  Quality of food intake (feeding ability)  View of participants (resident satisfaction)  **3º outcomes** | **Level of agitation (Pittsburgh Agitation Scale):**  72% didn’t have these behaviours at any point, 1 maintained a low level of motor agitation and 1 improved after being mildly aggressive. (no significant effects)  **Staff notes from focus group:** residents eating at a more leisurely pace, less wandering and more relaxed. | **E intake, kcal at lunch**  Baseline: 490 Kcal  Period 2 677 (p=0.6)  **Feeding Ability Assessment:** no effect  8/11 remained constant  1/11 dramatically improved  1/11 independent at baseline & wk6 but not wk12.  **Resident satisfaction: (n=8)** (on a scale of 1-4)  Baseline 3.13  Period 2 3.38  No effect |  |

**Supplemental Table 3. Characteristics and results of behavioural interventions (EDWINA)**

| **Study** | **Participants** | **Interventions** | **Outcomes*** | **1° outcomes*** | **2° outcomes*** | **3° outcomes*** |
| --- | --- | --- | --- | --- | --- | --- |
| **Beattie 2004**  **USA**  **Setting: Dementia specific unit/ Nursing home**  **Aim:**  To determine the effect of systematic use of behavioural nursing int on mealtime behaviour of wanderers. | **Participants**: NH dementia residents, current wanderers  **N:** 3  **M/F:** 1/2  **Mean age (SD)**: NR  **Nutritional status: mean BMI 20**  **Cognitive status:** MMSE score 0-1 **Dementia diagnosis: diagnosed (DSM-IV criteria)**  **Dementia type: AD**  **Dementia stage: Severe**  **Acute illness: No** | **Design: BA (multiple case study design)**  **Int Type:** Behavioural int  **Provided by: Trained intervener (researcher?)**  Details: systematic behavioural conditioning by trained intervener over 20 minutes evening meal. This involved reinforcement and minimal pressure by hand on shoulder then if resident left table, “grabbing” dominant arm and re-seating them.  **Cont**: no details  **Compliance:** NR  **Duration of int/ follow up**: 5 wks (of which 2wks of int wk 3 &5, 5 d each wk)  **Individualised: No**  **Ethics obtained: NR** | **1º outcomes**: Nutritional status (Weight)  **2º outcomes**:  Quantity of food intake (% of food/ drink consumed)  Frequency of table leaving events  Meantime sitting at the table  **3º outcomes** | **Body weight, lb: mean (SD) for 3 individuals**  **Case 1:** baseline 140.8 (1.7) vs int.140.1 (1.3)  **Case 2:** baseline 94.4 (0.7) vs int.95 (1.65)  **Case 3:** baseline 122.1 (1.7) vs int.122.2 (1.7)  **None statistically sig.** | **Food intake:** (% of food consumed), pooled results of daily observations, mean (SD)  Case 1: baseline 31 (20.8) vs int 31.3 (12.9)  Case 2: baseline 19.7 (11.8) vs int 41.2 (10.1) (p=0.05)  Case 3: baseline 13.7 (10) vs int 48.8 (22.1) (p=0.05)  **Fluid intake: (% of fluid consumed, pooled results of daily obs during both conditions), mean (SD)**  Case 1: baseline 42.3 (27.3) vs 56.8 (21.1) int  Case 2: baseline 26.6 (19.2) vs 46.2 (30.3) int  Case 3: baseline 66.3 (38.8) vs 83.1 (26.4) (none statistically signficant) |  |
| **Coyne 1988**  **USA**  **Setting**  **Dementia unit in nursing home**  **Aim:**  To determine the short and long term efficacy of directed verbal prompts and positive reinforcement on the level of eating independence of elderly nursing home patients with dementia. | **Participants**: Dementia unit residents  **N:** 24 (12 int. & 12 cont)  **M/F:** 0/24  **Mean age, SD**: 83.4 (9.1) int, 84.9 (7) cont,  **Nutritional status: not reported**  **Cognitive status: Short Portable Mental Status Q (SPMSQ)** (5-7 errors= moderate impairment, 7-10=severe)  Int. 9.4 (0.9), cont 9.7 (0.9) **Dementia diagnosis: DSM-III criteria**  **Dementia type: AD & others**  **Dementia stage: Severe**  **Acute illness: No** | **Design: RCT parallel**  **Int Type:** Verbal prompts  **Provided by: Researchers**  Details: Patients organised into groups of 4.  Directed verbal prompts & positive reinforcement administered when patients completed eating tasks at 1 min after food trays prepared & a standard rate of 1 min intervals.  **Cont**: Usual care  **Duration of int/ follow up**: 2 wks  **Compliance:** measured and indicates good compliance (no single figure to report, 3 pages of details)  **Individualised: No**  **Ethics obtained: Yes** | **1º outcomes**:  **2º outcomes**:  Level of eating independence for solid foods (scores of 8-20, 20 total independence, 8 total dependence & 12-16= partial independence),  Level of eating independence for liquid foods (scores of 7-16, 16 total independence, 7 total dependence & 10-13= partial independence)  Frequency of eating/ drinking)  **3º outcomes** |  | **Eating independence, solid foods, mean (SD)**  Int: Baseline 15.7 (3.5), 2 wks 16.5 (4.3)  Cont: baseline 13.6(3.8), 2 wks 12.6 (3.6)  P=0.011  **Eating independence for liquid foods:**  Int: Baseline 13.5 (2.3), 2 wks 13.5 (3.5)  Cont: baseline 11.7(1.7), 2 wks 10.9 (2.1)  ANOVA p=0.007 BUT no sig difference between groups for change.  **Frequency of eating solids, mean (SD)**  Int: Baseline 6.9 (2.2), 2 wks 7.8 (3.2)  Cont: baseline 7.9 (2.0), 2 wks 9.3 (2.0)  ANOVA P=0.06 (not sig)  **Frequency of eating liquid foods, mean (SD)**  Int: Baseline 1.7 (1.4), 2 wks 1.9 (1.5)  Cont: baseline 3.2(1.8), 2 wks 3.7 (1.8) ANOVA p=0.010 (sig effect at end, BUT not of change, baseline differences) |  |
| **Eaton 1986**  **USA**  **Setting**  **Skilled care facility**  **Aim:** To evaluate effects of gentle touch during eating on nutritional intake of institutionalized people with Chronic Organic Brain Syndrome. | **Participants**: self-feeding people with Chronic Organic Brain Syndrome  **N:** 42 (21 int., 21 cont)  **M/F:** NR  **Mean age (SD)**: int. 84.9(6.4), cont 85.4 (6.2)  **Nutritional status: NR**  **Cognitive status: NR** **Dementia diagnosis: NR**  **Dementia type: NR**  **Dementia stage: NR**  **Acute illness: NR** | **Design: RCT parallel**  **Int Type:** Gentle mealtime touch  **Provided by: Care workers**  Details: At mealtimes participants were touched lightly on the forearm while being verbally encouraged to eat. They were touched briefly for 5 times during each meal for a total of approx. 1 min in 1 hr period.  **Cont**: Normal verbal encouragement  **Duration of int/ follow up**: 5 d  **Compliance:** NR  **Individualised: No**  **Ethics obtained: NR** | **1º outcomes**: None  **2º outcomes**:  Quantity of food intake (energy intake, protein intake, E and protein consumed daily calculated 5 d pre-int, 5 d int & 5 d post int. in both groups)  **3º outcomes** |  | **E intake, kcal/d**  Int E intake higher than cont E intake during intervention and post-intervention weeks (p<0.05).  **Protein intake, g/d**  Int intake higher than cont intake during intervention and post-intervention weeks (p<0.05) |  |
| **Huang 2009**  **Taiwan**  **Setting**  **Older person care facility**  **Aim:**  To help older people with dementia in a home by applying reminiscence group work. | **Participants**: Nursing home dementia residents  **N:** 12  **M/F:** 7/4  **Age**: 4<74, 3=75-84, 4>85  **Nutritional status: NR**  **Cognitive status: MMSE, mean (SD)**  15.9 (5) **Dementia diagnosis: diagnosed (no details)**  **Dementia type: NR**  **Dementia stage: Mild to moderate**  **Acute illness: NR** | **Design: BA**  **Int Type:** Reminiscence therapy  **Provided by:** Researchers?  Details: 8 sessions of reminiscence cooking lessons of dishes remembered by the participants. A session was; 10 min warm up, 20 min cooking procedure and 30 min eating and sharing results. After each session participants were interviewed for 30 min.  **Cont**: N/A  **Duration of int/ follow up**: 8 wks  **Compliance:** NR  **Individualised: yes**  **Ethics obtained: NR** | **1º outcomes**:  Meaningful activity and/or enjoyment of food (Personal interaction scale (7 item scale include feeling of happiness, communication, talking, positive interaction, participation, and order and activity preference) - feeling of participation scale  **2º outcomes**:  Measure of cognitive status (MMSE)  **3º outcomes** | **Feeling of happiness subscale (mean, SD)**  Pre 4.6(0.92), post 5.8(0.4) p=0.01  **Positive communication subscale (mean, SD)**  Pre 4.7(1.19), post 5.6 (0.66) p=0.05  **Participatory Feeling Scale** (4 items include feeling of emotion, stress relief, adaptation and impression) all items improved except feeling emotions | **MMSE (mean, SD)**  Pre 15.9(5), post 17.1(4) (not significant) |  |
| **Lin 2010**  **Taiwan**  **Setting**  **Dementia unit**  **Aim:**  To investigate the effectiveness of spaced retrieval & Montessori-based activities in decreasing eating difficulties in older residents with dementia | **Participants**: residents of 3 special care dementia units with some eating difficulty (EdFED ≥2).  **N:** 85 (32 int 1 (SR), 29 int 2 (Montessori, 24 cont)  **M/F:** 40/45  **Mean age (SD)**: 81.8 (6.4) range 66-96  **Nutritional status: BMI, mean (SD)** SR 24.7 (4.3), Montessori 21.2 (3.4), cont 23.1 (2.7)  **Cognitive status: MMSE, mean (SD)** SR 13.6 (5.1), Montessori 10.8 (4.9), cont 10.5 (8.0) Range 0-23  **Dementia diagnosis: Diagnosed (from chart record)**  **Dementia type: NR**  **Dementia stage: various**  **Acute illness: NR** | **Design: RCT parallel**  **Int** Type:  Int 1: Spaced-retrieval,  Int 2: Montessori-based activities  **Provided by: research assistants**  Details: Ints were performed in 35-40 min sessions, 3 times/wk for 8 wks. The SR group received training consisting of two dimensions: eating procedure and eating behaviour. SR used immediate, 1, 2, 4, 8, 16, and 32 min time interval trials to train subjects. The  Montessori-based activity included only hand-eye coordination, scooping, pouring, and squeezing. This study added the matching and differentiating of edible and not-edible items to the program  **Cont**: routine activities  **Duration of int/ follow up**: 8 wks  **Compliance: NR**  **Individualised: No**  **Ethics obtained:** Yes | **1º outcomes**:  Nutritional status (weight, BMI, MNA)  **2º outcomes**:  Quantity of food intake (amount eaten)  Edinburgh Feeding Evaluation in Dementia (EdFED**,** higher scores indicate greater eating difficulty)  (Self-eating time, Physical assistance, verbal assistance, Fed by caregivers) outcomes presented in table but not clarified in methods or results text (the study concludes that the frequencies of physical assistance and verbal assistance for the  Montessori-based activity group after int were significantly higher than that of the cont  group, which suggests that residents who received Montessori-based activity need more physical and verbal assistance during mealtimes)  **3º outcomes** | **BMI (mean, SD)**  **Spaced-retrieval**  Baseline: 24.7 (4.3),  8 wks 24.8 (4.4)  **Montessori activity**  Baseline: 21.2 (3.4),  8 wks 21.1 (3.2)  **Cont:**  Baseline: 23.1 (2.7),  8 wks 23.1 (2.5)  **Weight (Kg, mean, SD)**  **Spaced-retrieval**  Baseline: 58.3 (9.4),  8 wks 58.2 (9.5)  **Montessori activity**  Baseline: 51.1 (7.8),  8 wks 51.0 (7.8)  **Cont:**  Baseline: 55.0 (8.6),  8 wks 54.9 (8.3)  **MNA (mean, SD)**  **Spaced-retrieval**  Baseline: 21.7 (4.3),  8 wks 24.0 (2.1)  p<0.01 compared to cont  **Montessori activity**  Baseline: 18.3 (3.9),  8 wks 18.0 (3.7)  p<0.01 compared to cont  **Cont:**  Baseline: 20.3 (2.2),  8 wks 20.3 (2.9) | **EdFED (mean, SD)**  **Spaced-retrieval**  Baseline: 4.3 (2.0),  8 wks 3.4 (1.9)  p<0.05 compared to cont  **Montessori activity**  Baseline: 5.0 (3.3),  8 wks 3.5 (1.8)  p<0.05 compared to cont  **Cont:**  Baseline: 5.1 (3.2),  8 wks 5.0 (0.2)  **Eating amount ( %, mean, SD)**  **Spaced-retrieval**  Baseline: 85.3 (11.4),  8 wks 90.7 (8.8)  **Montessori activity**  Baseline: 74.7 (22.9),  8 wks 78.4 (10.1)  P<0.05 compared to cont  **Cont:**  Baseline:79.0 (19.2),  8 wks 88.1 (17.8) |  |
| **Lin 2011**  **Taiwan**  **Setting: Dementia unit**  **Aim:** To investigate the efficacy of applying a Montessori int to improve the eating ability and nutritional status of residents with dementia in long-term care facilities. | **Participants**: residents of 2 dementia care units with some eating difficulty (EdFED ≥2).  **N:** 29  **M/F:** 17/12  **Mean age (SD)**: 82.9 (6.0)  **Nutritional status**  **Cognitive status: MMSE, mean (SD)** 11.2 (5.1) range 3-22 **Dementia diagnosis: diagnosed (no further details)**  **Dementia type: NR**  **Dementia stage: 3 mild, 14 moderate and 12 severe.**  **Acute illness: NR** | **Design: RCT crossover**  **Int Type:** Montessori-based activities  **Provided by: trainers**  Details: Montessori-based activity including hand-eye coordination, scooping, pouring, squeezing and matching (30min session x 3 sessions/wk for 8wk), followed by 2wk washout then 8wk routine activities.  **Cont**: routine activities  **Duration of int/ follow up**: 8wks (each group) plus 2wks washout  **Compliance: NR**  **Individualised: No**  **Ethics obtained: Yes** | **1º outcomes**:  Nutritional status (BMI, MNA)  **2º outcomes**:  Edinburgh Feeding Evaluation in Dementia (EdFED, measure of eating difficulty)  Eating time,  Eating behaviour scale (EBS, to assess eating functional ability)  (Also: self-feeding frequency, self-feeding time, verbal assistance, physical assistance, fed by caregivers)  **3º outcomes** | **Change in BMI (mean, SD)**  At 8wk: Int 0.26 (0.7),  cont 0.09 (0.9) p=0.25  **Change in MNA (mean, SD)**  At 8wk: Int -0.67 (1.9),  cont -1.33 (2.9) p=0.13 | **Change in EdFED score (mean, SD)**  At 8wk: Int –1.57 (3.4), cont -0.71 (2.4) p=0.008  **Change in Eating behaviour scale (EBS)**  **(mean, SD)**  At 8wk: Int 0.63 (2.8), cont -0.96 (3.0) p=0.03  **Change in Eating time (min, mean, SD)**  At 8wk: Int 1.1 (8.6),  cont -3.1 (9.4) p=0.07  No changes in need for verbal and physical assistance or needing to be fed by carers, but improvement in self-feeding frequency |  |
| **McHugh 2012**  **USA**  **Setting: Memory support unit/ care facility**  **Aim:** Will residents’ active engagement in singing lead to subsequent productive changes in nutritional intake? | **Participants**: Dementia patients residing in a memory support unit.  **N:** 15 (8 int., 7 cont)  **M/F:** 3/12  **Mean age**: 87.5 int., 86.3 cont.  **Nutritional status: NR**  **Cognitive status: MMSE (range 12-24)** mean 14.6 int., 19.7 cont **Dementia diagnosis: diagnosed**  **Dementia type: 13 AD, 2 other dementia**  **Dementia stage: mild to moderate**  **Acute illness: NR** | **Design: RCT parallel**  **Int Type:** Environment (Vocal re-creative music therapy)  **Provided by: music therapist**  Details: Residents taken to activity area 30min before lunch, seated in semi-circle with music therapist facilitator leading session. Range of songs (11 songs, lasted 25min) familiar to the age group, rated top 10 during decade of interest, slow to med tempo, range of genres, lyric form conducive to singing, able to be played on piano/guitar. Afterwards, residents taken to dining room, with routine care.  **Cont**: usual care  **Duration of int/ follow up**: 3wks (4d/wk)  **Compliance: NR**  **Individualised: No**  **Ethics obtained: Yes** | **1º outcomes**:  Meaningful activity  **2º outcomes**:  Quantity of food intake (proportion of food eaten)  **3º outcomes** | **Meaningful activity** (participation, based on video recording)  Some residents observed singing/ whistling fragments of session material during the meal.  No other mention of data from the video recording. | **lunch intake%:**  Int: baseline 70%, end: 73%  Cont: baseline 81%, end 86% (no statistical significance testing)  **6/8 participants in int. group increased intake while there was sharp decrease in 2/8.** |  |
| **Santo Pietro 1998**  **USA**  **Setting: Dementia unit within a nursing home**  **Aim:** To document the effectiveness of the breakfast club, a multi-modality group communication therapy for AD patients to compare its outcomes with those of standard conventional group therapy for institutionalised patients with mid-stage AD. | **Participants: Institutionalised patients with mid-stage dementia**  **N: 40 (20 int group)**  **M/F: not reported**  **Mean age (SD):**  **Int.84.6 (4.7), Cont 86.2 (6.03)**  **Cognitive status,**  **MMSE, mean (SD) Int. 15.6 (4.0), Cont (n=18) 13.8 (4.8)**  **Dementia diagnosis: NR**  **Dementia type: AD**  **Dementia stage: mild and moderate AD**  **Acute illness: NR** | **Design: CCT**  **Int Type:** multi-component (environmental improvements, social activities including food and drink, prompting)  **Provided by:** health worker (speech & language therapist)  **Details:** groups of 5 residents sat around a table and prepared, ate and cleared up breakfast, facilitated by researcher, who also encouraged conversation about the tasks.  **Cont:** groups of 5 residents sat around a table, coffee was served and conversation was facilitated by a researcher  Duration of int/ follow up: 45 minutes each morning/5 d per wk for 12 wks  **Compliance: NR**  **Individualised: No**  **Ethics obtained: NR** | **1º outcomes:**  **Interest & involvement** (‘a comment on an action or task at hand which is appropriate, unsolicited and self-initiated without prompt or cue’).  **Procedural memory score: (**‘demonstrated knowledge of how to perform familiar tasks and neuromotor memories that operate independently of informational knowledge’)  **2º outcomes:**  Measures of functional status, COMFI.  Measures of cognition, ABCD.  Views of participants, carers and family (anecdotal)  **3º outcomes: NR** | **Interest & involvement score:**  Int: 12.0  Cont: not reported  p<0.0005  **Procedural memory score:**  Int: 6.8  Cont: not reported  p<0.0005 | **COMFI, mean change:**  Int: 16.05 (SD: 8.22)  Cont: 0.5 (SD: 4.77)  p=<0.00001 (calculated by reviewers but numbers unlikely)  **ABCD.**  Int: 10.87 (SD: NR)  Cont: 10.25 (SD: NR);  **Views of participants, carers and family (anecdotal)**  Int: members remembered each other’s names; increased response to non-verbal cues, increased use of humour, use of empathetic statements, spontaneous singing; decreased distractibility and wandering behaviour. | **N/A** |
| **Van Ort 1995**  **Behavioural int**  **US**  **Setting: Secure nursing unit**  **Aim:** To test the efficacy of two nursing interventions, one contextual and one behavioural, designed to promote functional feeding and maintain adequate nutritional status of a sample of elders with dementia in long-term care settings. | **Participants**: ‘non-combative’ residents from a secure unit within a larger residential geriatric centre, who were able to sit in a chair for feeding, were responsive to human interaction, were not usually restrained for feeding, and required feeding assistance.  **N:** 7  **M/F:** 2/5  **Age range**: 65-93  **Nutritional status:** NR  **Cognitive status:**  Severe dementia, MMSE, range: 0-2  **Dementia diagnosis:** residents of dementia unit, method of diagnosis N/R  **Dementia type:** N/R  **Dementia stage:** severe  **Acute illness:** NR | **Design: BA**  **Int t**ype: behavioural  **Provided by: care staff**  Details:  **Behavioural int for 2 wks:**   - Use of verbal and tactile prompts - Role modelling - Cue synchronisation & reinforcement to elicit and sustain functional feeding (repetition, pantomiming of desired behaviour, tactile and verbal reinforcing of appropriate feeding behaviour)   **Cont**: N/A  **Duration of int/ follow up**: 10 wks (2 wks each int).  **Compliance:** NR  **Individualised: NR**  Behavioural int: yes  **Ethics obtained:** NR | **1º outcomes**:  Change in weight  **2º outcomes**:  Amount of food consumed and  Self-feeding behaviours.  **3º outcomes** | **Weight:** no change  Unclear whether any outcomes were statistically significant as no numbers presented (probably not given small sample size)  Suggestions that meal-times were extended, but there was increased independence**:**   - No change in weight - Greater food and drink intake - More frequent refusal of foods - Initiated eating more frequently - Touched food more often - Greater interaction with feeders |  |  |
| **Wu 2013**  **Taiwan**  **Setting: Dementia units**  **Aim:** To examine long-term effects on people with dementia of fixed and individualized spaced retrieval  combined with Montessori-based activities on nutritional status, BMI, and depressive symptoms | **Participants**: Dementia unit residents  **N:** 90 (fixed group= 25, Individual group= 38, cont= 27)  **M/F:** 90/0  **Mean age (SD)**: FG 82 (6.8), IG 83 (6.8), cont 83 (4)  **Nutritional status, BMI, mean (SD):** FG 20.3 (2.2), IG 22.3 (3.3), cont 22.9 (3.1)  **Cognitive status** MMSE **Dementia diagnosis:** Psychiatrist/ neurologist diagnosis  **Dementia type: NR**  **Dementia stage: 15 mild, 39 moderate, 36 severe.**  **Acute illness: NR** | **Design: CCT**  **Int Type:** Educational (spaced retrieval with Montessori activities)  **Provided by: Researcher**  Details: **Fixed therapy group** received spaced retrieval training combined with Montessori activities over 24 sessions.  **Individualised therapy group** received same int but with different sessions adjusted according to each participant’s learning response.  **Cont**: usual care  **Duration of int/ follow up**: NR (states 24 sessions over 8 wks for FG but 6 mo follow up period for all)  **Compliance:** NR  **Individualised:** yes (for individual group)  **Ethics obtained:** Yes | **1º outcomes:**  Nutritional status (BMI, Chinese-MNA)  **2º outcomes**  **3º outcomes** | **Fixed**   - Significantly greater BMI at 6 mo - Significantly lower nutritional risk at 6 mo - No change in depression at 6mo   **Individualised**   - Significantly greater BMI at 6 mo - Significantly lower nutritional risk at 6 mo - Significantly reduced depression at 6mo   (all assessed using linear mixed model analysis with time) |  |  |

**Supplemental Table 4. Characteristics and results of exercise interventions (EDWINA)**

| **Study** | **Participants** | **Interventions** | **Outcomes*** | **1° outcomes*** | **2° outcomes*** | **3° outcomes*** |
| --- | --- | --- | --- | --- | --- | --- |
| **FOPANU study (Carlsson 2011, Rosendahl 2006)**  **Sweden**  **Setting: 9 Residential care facilities**  **Aim:** To evaluate effects of high intensity functional exercise & timed protein/ energy drink on muscle mass | **Participants**: ADL dependent, cognitively impaired care homes residents  **N:** 191 randomised, 177 allocated (Exercise & Protein. 46, Exercise & placebo 45, contr ol &Protein 50, cont &placebo 50) (for protein 96 int vs 95 cont)  **M/F:** 52/139  **Mean age (SD)**: 84.5 (6.4)  **Nutritional status BMI, mean (SD)** 24.9 (4.6)  **Cognitive status**  MMSE, mean (SD) 17.6 (5.1) **Dementia diagnosis: NR 100/191 (52%) had dementia diagnosis but most were cognitively impaired based on MMSE**  **Dementia type: NR**  **Dementia stage: NR**  **Acute illness: NR** | **Design: RCT parallel (4 arms)**  **Int Type:** ONS (protein enriched drink plus/minus exercise)  **Provided by: care staff & health workers**  Details: High intensity functional exercise, 45 min/session, 29 sessions over 3mo, plus or minus high protein drink offered within 5 min of activity.  **Cont**: sitting activity, 45 min/session, 29 sessions over 13 wks, plus or minus high protein drink offered within 5 min of activity.  **Compliance**: protein drink was taken on 82% of occasions vs 78% for placebo  **Individualised: No**  **Duration of int/followup**: 3mo  **Ethics obtained: Yes** | **1º outcomes**:  Nutritional status (Weight)  Hydration status (Intra cellular water ICW)  **2º outcomes**:  Measures of functional status (Berg balance scale, gait speed, lower limb strength)  **3º outcomes**  Mortality | **Body weight, kg, mean (SD) at 6mo**  [Exercise ± protein]-[Control activity ± protein] = -1.3 (95%CI -2.3 to -0.20), p=0.021  Lower in exercise gp  **Intra cellular water (ICW), L, mean (SD) at 6mo**  [Exercise ± protein]-[Control activity ± protein] = -0.5 (95%CI -0.9 to -0.02), p=0.039  Lower in exercise gp | **Balance at 6mo**  p=0.05  **Gait speed, self-paced, m/s, at 6mo**  p=0.009  **Gait speed, maximum, m/s, at 6mo**  p=0.30  **Lower limb strength, kg, at 6mo**  p=0.03 | **Mortality at 6 mo**  Exercise ± protein: 7/91  Control activity ± protein: 3/100  p>0.05 |
| **Chang 2011**  **Taiwan**  **Setting: Day care centre**  **Aim:** To examine the effectiveness of an exercise program for elders with dementia in Taiwan aimed to maintain their activities of daily living. | **Participants**: Taiwanese dementia patients attending day centre  **N:** 29 (26 analysed)  **M/F:** 8/18  **Mean age (SD)**: 75.2 (5.8)  **Nutritional status:** NR  **Cognitive status**   **Dementia diagnosis:** Physician diagnosis  **Dementia type:** NR  **Dementia stage:** NR  **Acute illness:** NR | **Design: BA**  **Int Type:** Exercise programme  **Provided by:** day centre care workers.  Details: Exercise programme consisting of: 5 min stretching & walking, 5 times/wk plus 20-30 min leg weight-bearing, 3 times/wk. Encouragement and rewards given.  **Cont**: N/A  NR whether compliance was measured or not.  **Duration of int/follow up** 4mo  **Compliance:** NR  **Individualised: No**  **Ethics obtained: Yes** | **1º outcomes**: None  **2º outcomes**:  Ability to eat independently (ADL feeding, **score 0-126, higher score= greater independence)**  ADL performance score **(score 0-780, higher score= greater independence)**  Changes in physical fitness  **3º outcomes** |  | **ADL Feeding**  **Changes in ADL feeding:**  **2 mo** p=0.382  **4mo** p>0.05  **ADL performance, mean (SD)**  **Baseline:** 546.8 (60.1)  **2 mo.:** 609.1 (49.5) P<0.001 (compared to baseline)  **4 mo.:** 588.7 (61.7) P=0.042 (compared to baseline) |  |
| **Dechamps 2010**  **France**  **Setting: 3 NHs & 1 Long term care home**  **Aim:** To assess the effects of targeted exercise programs on health-related quality of life compared with usual care based on ability to perform ADL and the Neuro Psychiatric Inventory scores in geriatric institutionalised persons | **Participants**:  **N:** 160 (AT 51, CA 49, cont 60)  **M/F:** 45/ 115  **Mean age (SD) AT 83 (8.6), CA 83 (8.3), cont 81 (10.1)**:  **Nutritional status: MNA, number with score <17 (%) AT 9 (18.4), CA 7 (16.3), cont 20 (35.7)**  **Cognitive status: MMSE** AT 17.3 (9.3), CA 14.2 (9.3), cont 14.8 (7.8) **Dementia diagnosis: 94 (59%) diagnosed with diagnosis retrieved from records or confirmed by researchers. (however, most are cognitively impaired based on MMSE)**  **Dementia type: AD & others**  **Dementia stage: NR**  **Acute illness: NR** | **Design: RCT parallel (3 arms)**  **Int Type:** Exercise programs  **Provided by: trained staff**  Details: **Adapted tai chi (AT),** emphasized body sensation, awareness of weight shifting, body alignment, and coordination with deep breathing and muscle exercises (4x 30mins/wk, 6mo)  **Cognition-action (CA)** 10-minute warm-up while seated in circle, lower limb movements alternated with upper body exercises, followed by stretching and resistance exercises, balance exercises, passing balls to neighbours, then deep-breathing and relaxation (30-40mins,2x/wk for 6mo)  **Cont**: usual care  **Compliance**: measured 48.9% (29.8) attendance rate for CA & 38.8% (32.3) for AT  **Individualised: No**  **Duration of int/ follow up**: 6 mo & 12 mo follow up  **Ethics obtained: Yes** | **1º outcomes**: None  **2º outcomes**: Ability to eat independently (ADL eating subscale) **(eating independence a scale of 0-2 with 0 eating with no help to 2 unable to eat without complete help),**  Measures of cognitive status (MMSE)  **3º outcomes**  **Additional outcomes:** Measures of functional status (ADL score, Hand Grip Strength, chair rise test, Timed Up & Go)  Total and subscales of Neuropsychiatric Inventory (NPI) |  | **ADL, Eating sub-scale** adjusted mean difference (SD)  **Baseline:** AT 0.3 (0.6), CA 0.5 (0.6), cont 0.5 (0.7)  **Change at 12 mo:** AT -0.1 (0.63), CA 0.1 (1.0), cont 0.38 (0.89**)**  P=0.001 suggesting improvements in both int groups  **MMSE** (adjusted mean difference score, SD)  Change at 12 mo: AT -0.75 (10.7), CA -0.75 (8.5), cont -1.64 (7.3)  p=0.21, no effect in either int group |  |
| **FICSIT trial**  **Fiatarone 1994**  **USA**  **Setting**  **Nursing home (long term rehab centre)**  **Aim:**  To compare progressive resistance exercise training, multinutrient supplementation, both ints, and neither in 100 frail nursing home residents over a 10-wk period | **Participants**: Institutionalised elders  **N:** 100 (25 exercise(1), 25 ex plus supplement(2), 24 supplement(3), 26 cont)  **M/F:** 63/100  **Mean age (SD)**: 87.1 (6) range 72-98)  **Nutritional status: BMI, mean (SD)** *(1)* 24.9(3.5), *(2)* 24.5 (4), *(3)* 25.4 (3.4), cont 25.6 (0.5)  **Cognitive status: MMSE, mean (SE)** *(1)* 20.9(1.2), *(2)* 23.1 (1.0), *(3)* 22.7 (1.3), cont 22.2 (5.1) - 51% had MMSE<24 **Dementia diagnosis: NR**  **Dementia type: NR**  **Dementia stage: NR**  **Acute illness: No** | **Design: RCT parallel (4 arms)**  **Int Type:** Exercise ± ONS  **Provided by: exercise trainer**  Details: high-intensity exercise - progressive resistance of hip and knee with weight machines supervised individually (3 x 45 minutes sessions/wk) ± ONS (1x240 ml liquid supplement/d for 10 wks offered in a choice of flavours)  **Cont**: Placebo activities ± ONS  **Duration of int/ follow up**: 10wk  **Compliance:** 97% with exercise sessions, 99% with supplement, 100% for placebo drink and activities  **Individualised: No**  **Ethics obtained: Yes** | **1º outcomes**:  Nutritional status (Weight, Thigh-muscle area)  Hydration status (whole body potassium)  **2º outcomes**:  Quantity of food intake (energy intake)  Measures of functional status (Muscle strength and mobility) | **Weight, kg**  Exercise ± ONS vs placebo activities ± ONS p=0.19  **Thigh muscle area, cm^2^**  Exercise ± ONS vs placebo activities ± ONS p=0.14  **whole body potassium, g**  Exercise ± ONS vs placebo activities ± ONS p=0.79 | **E intake (kcal/d, mean, SD)**  “Augmentation of E intake due to exercise”, p<0.01  **Physical activity** (count, after adjustments) “significant increase” in exercise ± ONS vs placebo activities ± ONS BUT p=0.12  **Muscle strength and mobility** (various measures inc stair climbing power all increased w exercise, each p<0.05) | **Mortality**  Ex ± ONS 0/50  Control ± ONS 2/50  p>0.05 |
| **Heyn 2003**  **USA**  **Setting: Memory care residence**  **Aim:** To evaluate the outcomes of a multisensory exercise program on cognitive function, behaviour & physiological indices in nursing home residents diagnosed with moderate to severe dementia | **Participants**: ambulatory AD patients at a memory clinic  **N:** 13  **M/F:** 1/12  **Mean age (SD)**: 85.7 (6.5) range 70-93  **Nutritional status**  **Cognitive status: MMSE, mean (SD)** 7.25(3.4)  **Dementia diagnosis: NINCDS-ADRDA criteria**  **Dementia type: AD**  **Dementia stage: mostly severe**  **Acute illness: No** | **Design: BA**  **Int Type:** Exercise program  **Provided by**: health workers (exercise physiologist with training in gerontology)  Details: Multisensory exercise program with 4 components; focused attention & warm up, flexibility & aerobic exercise, a strength training and relaxation & breathing techniques (15-70mins, after lunchtime 3x/wk for 8 wks).  **Cont**: N/A  **Duration of int/follow up**: 8wk  **Compliance:** 69.2% engaged in > half of the activity, 30.8% engaged in up to half.  **Individualised: No**  **Ethics obtained: NR** | **1º outcomes**:  Nutritional status (weight)  **2º outcomes**:  Engagement **(measured using Menorah Park Engagement Scale (MPES)**, completed by 8 examiners (family members, carers & activity directors) (1-3 with 3=engaged >1/2 & 1= not engaged)  Mood **measured using (Caregiver Report questionnaire CMR)**  **3º outcomes** | **Weight, lb (mean (SD))**  Baseline 121 (17.8), at 8 wks 121 (16.7) change 0.3 (3) | **Engagement**  Baseline 30% engaged, post int. 70% engaged.  **Overall mood:**  at 8 wks 61.5% showed mood improvement (compared to 38.5% with no or little improvement) |  |
| **Moore 2010**  **USA**  **Setting: Nursing home and assisted living facility**  **Aim:** To examine whether physical activity to familiar music would reduce apathy and agitation and increase eating ability and dietary intake among older adults with dementia. | **Participants**: Institutionalised older adults with dementia  **N:** 84 (43 int., 41 cont)  **M/F:** 18/66  **Mean age (range)**: 85.9 (68-99)  **Nutritional status: NR**  **Cognitive status: MMSE, mean (range) 12.3 (0-24)** **Dementia diagnosis:**  **Dementia type: 24 AD, 6 vascular, 5 mixed and 49 not specified**  **Dementia stage: Various**  **Acute illness: No** | **Design: RCT parallel**  **Int Type:** Exercise and music  **Provided by: Researcher**  Details: Seated chair exercise choreographed to familiar music of 1920s-1950s (for 30 min before mealtimes, 2x/wk for 3 wks)  **Cont**: quiet activity, no music and in some cases waited in the dining room for meal  **Duration of int/follow up**: 3wk  **Compliance: Not measured**  **Individualised: No**  **Ethics obtained: Yes** | **1º outcomes**:  **2º outcomes**:  Quantity of food and fluid intake  Eating ability-Functional independence **(eating subscale of ADL** (1= complete dependence to 7 complete independence)  **3º outcomes**  **Other outcomes include agitation & participation** |  | **Total food and fluid intake, %**  Int: 76.7%,  Cont 61.6%  p=0.01  **Eating ability**  Int: baseline 5.1 (1.1), end 5.6 (1.5)  Cont: baseline 4.8 (1.2), end 5.7 (1.1)  **p>0.05** |  |
| **Rolland 2007**  **France**  **Setting: Nursing home**  **Aim:** To investigate the effectiveness of an exercise program in improving ability to perform ADL, physical performance and nutritional status and decrease behavioural disturbances in patients with AD. | **Participants**: Residents with AD from 5 nursing home.  **N:** 134 randomised (67 int., 67 cont) 110 analysed (56 int., 54 cont)  **M/F:** 33/101  **Mean age (SD)**: 82.8 (7.8) int., 83.1 (7.0) cont.  **Nutritional status: MNA, mean (SD)** int. 22.2 (3.1), cont 21.8 (2.6).  **Cognitive status: MMSE, mean (SD)** int. 9.7 (6.8), cont 7.9 (6.4). **Dementia diagnosis: NINCDS-ADRDA criteria**  **Dementia type: AD**  **Dementia stage: mild to severe.**  **Acute illness: Yes** | **Design: RCT parallel**  **Int Type:** Exercise  **Provided by: occupational therapist**  Details: Exercise program including aerobic, strength, flexibility, and balance training, plus walking (1hr in afternoon 2d/wk, for 88 sessions).  **Cont**: Usual care  **Duration of int/ follow up**: 12 mo  **Compliance:** Of the 56 exercisers who completed the study, mean adherence  was 33.2% (25.5)% of the 88 sessions  **Individualised: Yes** (Exercise groups of 2-7 people selected according to cognitive and functional status and friendships, exercise selected for each group individually)  **Ethics obtained:** Yes | **1º outcomes**:  Nutritional status (weight, MNA)  **2º outcomes**:  Functional status (ADL **Katz score,** 0-6,  0= dependent &  6= independent)  **3º outcomes** | **Weight, mean (SD)**  Int Baseline: 62.4 (16.2), 1year 61.9 (15.7)  **Cont:**  Baseline: 60.6 (12.7), 1 year 59.5 (12.5)  P=0.51  **MNA, mean (SD) Int**  Baseline: 22.2 (3.1), 1 year 20.7 (3.4)  **Cont:**  Baseline: 21.8(2.6), 1 year 20.4 (4.7)  P=0.16 | **ADL** mean (SD)  Int  Baseline: 3.2 (1.3), 1 year 2.6 (1.5)  Cont:  Baseline: 3.1 (1.3), 1 year 2.2 (1.5)  P=0.02 |  |

**Supplemental Table 5. Characteristics and results of multicomponent interventions (EDWINA)**

| **Study** | **Participants** | **Interventions** | **Outcomes*** | **1° outcomes*** | **2° outcomes*** | **3° outcomes*** |
| --- | --- | --- | --- | --- | --- | --- |
| **Beck 2010**  **Denmark**  **Setting: Nursing home**  **Aim:** To test the hypothesis that a multifaceted 11 wk int would have a significant influence on nutrition and function in elderly nursing home residents | **Participants**: elderly NH residents  **N:** 121 randomised (int 62, cont 59), 109 analysed? (int 54, cont 55)  **M/F:** 33/88  **Age, median (95% CI)**:87 (84-90) int, 86 (84-87) cont  **Nutritional status: BMI , median (95% CI):** int 23.4 (21.8-24.8), cont 23.4 (21.3-25.2)  **Cognitive status:** Cognitive performance scale, median (95% CI): int 3 (2-5), cont 3 (2-3) (scale of 0-6 with 0 intact & 6 v severe impairment) **Dementia diagnosis: NR**  **Dementia type: NR**  **Dementia stage: NR**  **Acute illness: NR** | **Design: RCT parallel**  **Int Type:** Multifaceted (nutrition, exercise & oral care)  **Provided by:** Care home staff & health workers  **Details: Nutrition**: 25g chocolate + 150 hot choc or 150 ml homemade suppl/ d + 150 ml suppl provided after exercise twice/wk. Gratin diet for those with swallowing difficulties.  **Exercise:** moderate intensity individualised (40-60 min 2x/wk, in groups of 2-5) supervised by physiotherapists.  **Oral care**: offered 1-2/wk by dental hygienist to reduce plaque.  **Cont**: Usual care  **Duration of int/ follow up**: 11 wks/ 27 wks  **Compliance:** NR, excluded non-compliant participants.  **Individualised: Yes**  **Ethics obtained: Yes** | **1º outcomes**: Nutritional status (BMI, Weight)  **2º outcomes**: Quantity of food intake (Energy intake, protein intake)  Measures of functional status (ADL, Berg’s balance scale, hand grip strength)  Measures of cognitive status (MDS-CPS)  **3º outcomes**  Mortality | **% Change in BMI, median (95% CI), 11wk**  Int: 0.4 (0.0-1.0)  Cont -0.2 (0.0-0.0) (p=0.003)  **%Change in Body weight, median (95% CI) at 11 wks**  Int: 1.3 (0.6-3.2)  Cont: -0.6 (-1.6-0.6) (p=0.005)  No difference from 11- 27 wk follow up, p=0.91 | **Change in E intake, MJ/d, median (95%CI), 11wk**  Int 0.7 (-0.3-1.2)  Cont -0.3 (-0.7-0.3) (p=0.08)  **Protein intake change, g/d, med (95%CI), 11wk**  Int 5 (-1-10)  Cont -2 (-5-4) (p=0.01)  **Change in ADL, mean score (95%CI), 11wk**  Int -0.1 (-1.3-1.07)  Cont -0.8 (-1.8-0.2) (p=0.26) (also no sig change from 11 to 27 wk)  **Change in CPS, mean (95%CI), 11wk**  Int 0.2 (-1.1-0.5)  Cont 0.0 (-0.3-0.3) (p=0.25) (also no sig change from 11 to 27 wk) | **Mortality 0-27 wk, n/N**  Int 15/62  Cont 8/59  (p>0.05) |
| **Boffelli 2004**  **Italy**  **Setting: Dementia unit**  **Aim:** To evaluate nutritional changes with 6 & 18 mo of follow up after a nutritional int program. | **Participants**: Malnourished dementia unit patients  **N:** 19 (40 screened for malnourishment, characteristics provided for 40)  **M/F:** 11/29  **Mean age (SD)**: NR  **Nutritional status:** 47.5% (19/40) malnourished albumin <3.5 g/L  **Cognitive status**  mean MMSE score (SD) 5.1 (5.9) **Dementia diagnosis: diagnosed (no details)**  **Dementia type:** 31 AD, 4 VD, 4 mixed & 1 Lewy bodies.  **Dementia stage: Severe**  **Acute illness: NR** | **Design: BA**  **Int Type:** Nutritional program  **Provided by: health worker? NR**  Details: diet composition, quality & consistency modified on preference, swallowing ability, dental status, increased feeding time & assistance, dining environment modified, ONS prescribed for low intake.  **Cont**: N/A  **Duration of int/ follow up**: 18 mo  **Compliance:** Not measured  **Individualised: Yes**  **Ethics obtained: NR** | **1º outcomes**: Nutritional status (BMI, Weight, serum albumin, cholesterol, haemoglobin, iron binding capacity)  **2º outcomes**:  **3º outcomes** | **Body weight, kg, mean (SD)**  Baseline: 58.5 (11.7)  6 mo: 57.7 (11.2)  18 mo: 61.1 (12.5)  (p=0.5)  **BMI, mean (SD)**  Baseline: 22.7 (3.2)  6 mo: 22.8 (3.7)  18 mo: 23.7 (3.4)  (p=0.35)  **Serum albumin, g/dL, mean (SD)**  Baseline: 3 (0.3)  6 mo: 3.5 (0.3)  18 mo: 3.4 (0.3)  (p<0.05)  **No. malnourished** (based on albumin)  Baseline: 19/40  6 mo: 10/40 |  |  |
| **Keller 2003**  **Canada**  **Setting: 4 Special care units in 2 long term care facilities**  **Aim:** To determine whether body weight can be maintained or improved in dementia residents of special care units using a comprehensive int strategy. | **Participants**: patients with dementia and orally fed from 4 special care units  **N:** 82 (33 int., 49 cont)  **M/F:** 31/51  **Mean age (SD)**: 79.7(7.2) int., 79.8(7.4) cont  **Nutritional status: BMI, mean:** int. 24.9 M & 24.8 F, cont 23.9 M& 25 F  **Cognitive status: MMSE, mean (SD)** int.9(5.9), cont 8.3(6.6)  **Dementia diagnosis: diagnosed (dementia unit)**  **Dementia type: AD (84.8% int. & 69.4% cont) and others**  **Dementia stage: NR**  **Acute illness: NR** | **Design: CCT**  **Int site**: Type: food service & staffing (comprehensive int)  **Provided by: Care home staff & dietitian**  Details: Enhanced dietitian time and enhanced menu:  * 1^st^ 9mo = standard care as cont.  * 2^nd^ 9mo: dietetic time enhanced as needed, menu modified (portable high-energy snacks, super-mashed potato as a standard, high protein milk), changes to high protein/ energy diet, increased awareness and nutrition communication.  * following 12mo: dietitian returned to standard, enhanced menu cont  **Comparison sites**: standard nutritional care menu; 3 meals and 2 snacks. Dietetic time 15min/ resident/mo.  **Duration of int/ follow up**: 30 mo (9mo baseline, 9mo int & 12mo follow up)  **Compliance: NR**  **Individualised: yes**  **Ethics obtained: Yes** | **1º outcomes**:  Nutritional status (weight)  **2º outcomes**:  Dietitian time, hospital d  **3º outcomes**  Mortality, infections, | **Weight change over 30 mo, %, mean (SD)**  Int. +4.8% (0.7%), Comparison -4.5% (0.9%)  p<0.001  Int: 27.3% gained >5% body weight & 6.1% lost>5%  Comparison: 6.8% gained >5% & 36.4% lost>5% | **Dietetic time, mins, mean (SD) (for 9 mo int)**  Int: 533.3 min (94.5)  Comparison: 17.6 (21.6)  p<0.001  **Hospital d (mean, SD) (for 9 mo int)**  Int: 0.85 (2.9)  Comparison: 0.4 (1.3)  p>0.05 | **Mortality: %**  Int: 6.1%  Comparison: 16.3%  p>0.05  **Infections (mean, SD):**  Int: 2.3 (1.6)  Comparison: 2.6 (1.7)  p>0.05 |
| **Simmons 2001**  **USA**  **Setting: Two Nursing Homes**  **Aim:** To evaluate a three-phase behavioural int to improve fluid intake in nursing home residents. | **Participants**: incontinent residents from two community nursing homes  **N:** 63 (48 int)  **M/F:** Int: 4/44; Cont: 5/10  **Mean age (SD):** Int. 88.7 (7.1); Cont 86.3 (6.1)  **Nutritional status, total mealtime food & fluid intake, % (SD) of portion served:**  Int.53.8 (15.6); Cont: 58.9 (14.7)  **Nutritional status (n=63), between-meal fluid intake, oz/d, (n=50):** Baseline data N/R  **Hydration status (n=32), serum osmolality, mOsmol/kg, mean (SD):**  Int: 303.6 (9.1); Cont: 303.4 (8.5).  **BUN:creatinine ratio**, mean (SD):  Int: 21.7 (6.1); Cont: 24.0 (4.6)  **Cognitive status**, MMSE, mean (SD): Int. 12.1 (7.9); Cont 13.9 (6.5).  **Dementia diagnosis:** Diagnosis of dementia or MMSE score (assessed by researchers)  **Dementia type:** NR  **Dementia stage:** NR  **Acute illness:** NR | **Design: CCT**  **Int**: Behavioural  **Provided by:** Research Staff  **Details:** Wks 1-16 (phase 1): residents prompted to exercise every two hours (7.00am–3.30pm) and checked for incontinence, offered toileting assistance and drinks.  Wks 17-24 (phase 2): increased to eight prompts/d  Wks 25-32 (phase 1): this was supplemented by an increased choice of drinks  **Cont**:  Usual care (not described)  **Duration of int/ follow up**: 32 wks  **Compliance:** Phase 1: mean number of prompts (both groups combined) = 3.8/d (SD: 0.2); Phases 2&3: 7.6/d (SD: 0.4)  **Individualised:** No  **Ethics obtained:** Yes | **1º outcomes (n=17 int, 15 cont)**:  Hydration Status: serum osmolality, BUN:creatinine ratio  **2º outcomes**:  Quantity of fluid intake  **3º outcomes**  Mortality | **Serum osmolality, mOsmol/kg, mean (SD):**  Int: 297.0 (10.8);  Cont: 294.7 (11.9). *p*=0.57  **BUN:creatinine ratio, mean (SD):**  Int: 22.9 (5.6);  Cont: 23.8 (7.2), p=0.71  *p values calculated by reviewers* | **Total mealtime food & fluid intake, % (SD) of portion served:**  ***Phase 1, assessed at 8 wks:***  Int: 52.0 (17.5);  Cont: 58.8 (15.6)  ***Phase 3, assessed at 32 wks:***  Int: 53.1 (19.6);  Cont: 57.3 (17.7)  *p=0.43 (calculated by reviewers)*  **Between-meal fluid intake, oz/d:**  ***Phase 1:***  Int: 9.8 (4.6);  Cont: N/R  ***Phase 2:***  Int: 16.1 (10.0);  Cont: N/R  ***Phase 3:***  Int: 21.4 (12.7);  Cont: N/R |  |

**Abbreviations:**

BA= before after study

CCT= controlled clinical trial (non-randomised)

cont= control

d=day

EDWINA= Eating and Drinking Well IN dementiA

int=intervention

mo=month

NR= not reported

RCT= randomised controlled trial

wk= week

*Primary, secondary and tertiary outcomes according to the EDWINA protocol

**Primary outcomes**

- Nutritional status (e.g. body mass index, weight, or any recognised nutrition marker)
- Hydration status (e.g. plasma osmolality, tonicity or osmolarity, urine volume, osmolality or specific gravity, admission to hospital with acute dehydration or acute kidney injury, or provision of intravenous or subcutaneous fluids)
- Meaningful activity and/or enjoyment of food and/or drink (activity around food or drink that is personally fulfilling, that people enjoy, look forward to or find important)?
- Measures of quality of life

**Secondary outcomes**

- Quantity of food intake (e.g. proportion of food provided that is eaten, energy intake)
- Quantity of fluid intake (e.g. volume of drinks imbibed daily)
- Quality or adequacy of food and/or drink intake (including ability to eat independently, and ability to swallow without aspirating)
- Measures of functional status (e.g. Barthel Index, Activities of Daily Living, mobility)
- Measures of cognitive status (eg mini-mental state exam)
- Views or attitudes of participants, carers and staff
- Cost effectiveness measures, or measures related to resource use (such as unscheduled hospital admissions)

**Tertiary outcomes**

- Mortality
- Health outcomes such as urinary tract infections, kidney stones, constipation, measures of continence, wound healing, respiratory infection, aspiration pneumonia, other infections (that may be related to nutrition or hydration status)

Included studies had to assess at least one of the outcomes marked with a diamond.

**Supplemental Figure 1. Risk of bias summary for dining environment and food service interventions**

Note: These are the criteria used to assess the elements of validity within included studies:

- Random sequence generation: Was there selection bias (biased allocation to interventions) due to *inadequate generation of a randomised sequence*?
- Allocation concealment: Was there selection bias (biased allocation to interventions) due to *inadequate concealment of allocations prior to assignment*?
- Blinding of participants and personnel: Was there performance bias due to knowledge of the allocated interventions by participants and personnel during the study?
- Blinding of outcome assessment: Was there detection bias due to knowledge of the allocated interventions by outcome assessors?
- Incomplete outcome data: Was there attrition bias due to amount, nature or handling of incomplete outcome data?
- Selective reporting: Was there reporting bias due to selective outcome reporting?
- Funding of study: Was there bias due to commercial funding/ involvement?
- Dementia or MCI diagnosis: Was there bias due to dementia/ mild cognitive impairment diagnosis not reported in accordance with recognised criteria?
- Baseline comparability: Was there bias due to significant baseline differences between study groups?
- Outcomes measured: Was there bias due to selected outcome measures not being suitable to reflect an improvement?

**Supplemental Figure 2. Risk of bias summary for educational interventions**

**
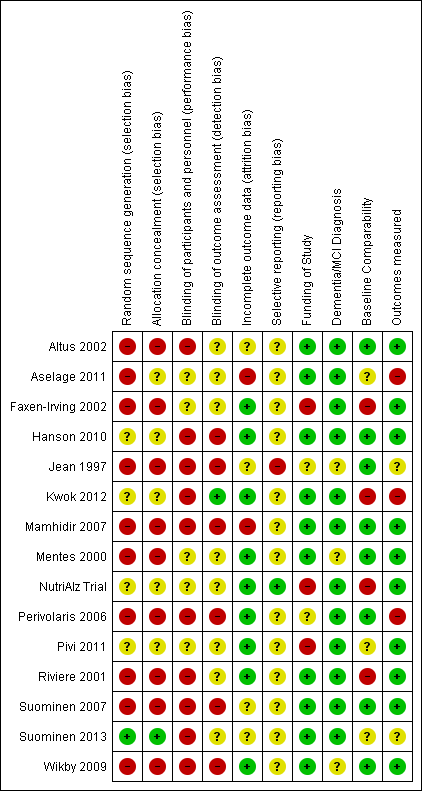
**

**Supplemental Figure 3. Risk of bias summary for 12 behavioural interventions**

**
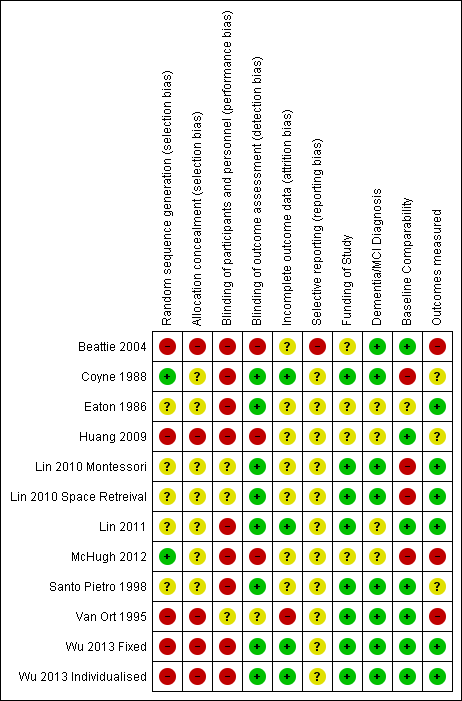
**

**Supplemental Figure 4. Risk of bias summary for exercise interventions**

**
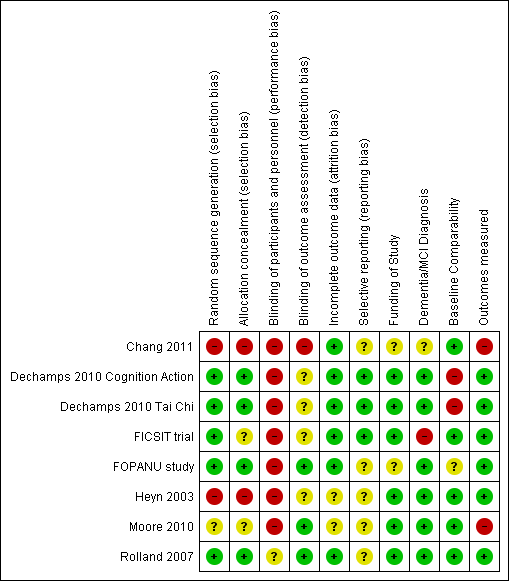
**

**Supplemental Figure 5. Risk of bias summary for multicomponent interventions
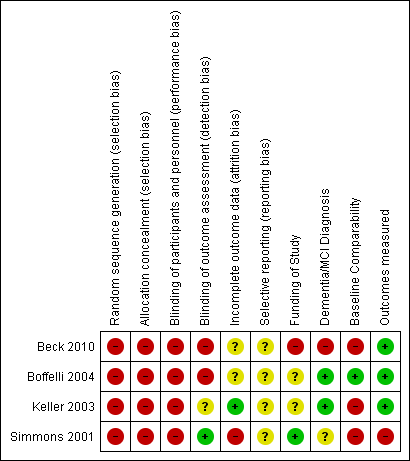
**
